# The Role of Bridge-State Intermediates in Singlet Fission for Donor–Bridge–Acceptor Systems: A Semianalytical Approach to Bridge-Tuning of the Donor–Acceptor Fission Coupling

**DOI:** 10.1021/acs.jpclett.1c03700

**Published:** 2022-01-20

**Authors:** Stephanie Valianti, Spiros S. Skourtis

**Affiliations:** † Department of Physics, University of Cyprus, 1678 Nicosia, Cyprus

## Abstract

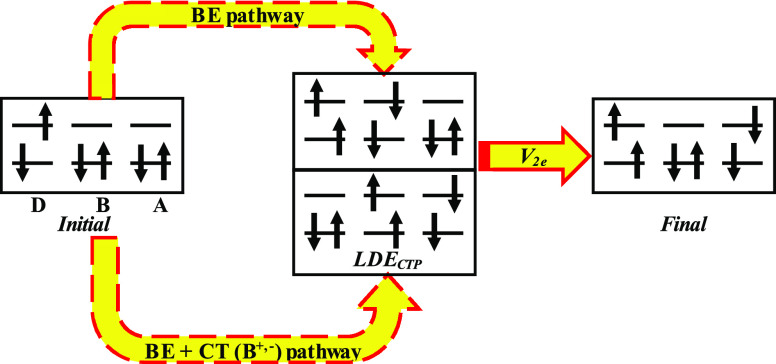

We describe a semianalytical/computational framework to explore structure–function relationships for singlet fission in Donor (D)–Bridge (B)–Acceptor (A) molecular architectures. The aim of introducing a bridging linker between the D and A molecules is to tune, by modifying the bridge structure, the electronic pathways that lead to fission and to D–A-separated correlated triplets. We identify different bridge-mediation regimes for the effective singlet-fission coupling in the coherent tunneling limit and show how to derive the dominant fission pathways in each regime. We describe the dependence of these regimes on D–B–A many-electron state energetics and on D–B (A–B) one-electron and two-electron matrix elements. This semianalytical approach can be used to guide computational and experimental searches for D–B–A systems with tuned singlet fission rates. We use this approach to interpret the bridge-resonance effect of singlet fission that has been observed in recent experiments.

Singlet Fission (SF) is a spin-allowed process in which a photoexcited singlet state *S*_1_ in a molecular system is converted into two correlated triplet excited states 2 × *T*_1_.^1−4^ SF offers a promising way to overcome the Shockley–Queisser limit on the efficiency of single-junction photovoltaics,^1−3,5^ and it is possible if a material satisfies the exoergicity criterion Energy (*S*_1_) ≥ 2 Energy (*T*_1_). An extensive body of work has examined SF in different materials due to its potential for photovoltaics applications.^6−13^ There are many experimental, computational,^14−17^ and theoretical studies^3,4,14,18,19^ of SF mechanisms and of the roles of SF intermediate states in dimeric systems (donor (D)–acceptor (A)). More recently, systems in which a bridge (B) unit connects the D and A moieties^11−13^ have received attention with the purpose of understanding how a bridge linker mediates SF.^5,20−26^ Recent experiments^24−26^ have observed a correlation between SF-rate enhancement and the lowering of the B HOMO–LUMO gap (the “bridge-resonance effect” of SF^26^). Another important direction in the field is the computational design of SF molecular assemblies with tunable SF rates.^27−31^

In this paper, we introduce an analytical framework, supported by ab initio computations, to explore structure–function relationships for bridge-mediated SF in D–B–A molecular architectures. The aim of this type of analysis is to understand how the bridge affects the SF coupling based on parametrized analytical models, and ultimately to guide the synthesis of D–B–A systems for tuned SF. We apply our method to bridge-mediated SF in the coherent tunneling regime. We also use our analytical results to interpret the recent experiments on bridge-resonance effects of SF.

Consider a D–B–A molecular assembly to be used as a tunable singlet-fission/triplet-separation device. Suppose that initial photoexcitation leads to a singlet excited state localized on the D moiety that subsequently undergoes SF to create a Correlated Triplet-Pair (CTP) state where one triplet is localized in the D moiety and the other is localized in the A moiety. In the coherent tunneling regime, where all SF intermediates are off(quasi)-resonant to the initial and final states, the SF rate is given by *k*_SF_ = |*V*_SF_|^2^ρ_FC_, where *V*_SF_ is the bridge-mediated effective SF coupling between initial (D singlet) and final (D–A CTP) states, and ρ_FC_ is the Franck–Condon factor. Therefore, the efficiency of D–A CTP creation can be tuned by controlling *V*_SF_ through structural modifications of the bridge. To this end it is necessary to obtain structure–function relationships for the bridge-mediated SF pathways that contribute to *V*_SF_.

The electronic Hamiltonian operator of the system is given by *Ĥ*^el^ = *ĥ*^1e^ + *V̂*^2e^, where
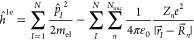
 is the Hamiltonian for *N* independent electrons in the field of *N*_nuc_ atomic nuclei and
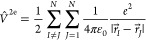
 is the total electron–electron Coulombic operator. In our modeling, we use a D, B, A fragment-orbital basis set to describe many-electron states for the D–B–A system.^32−34^ This is a natural representation since any approach to the design of SF molecular assemblies is necessarily fragment-based. We construct the many-electron basis to represent *Ĥ*^el^ using the Configuration Interaction method with single and double excitations (CISD).^35,36^ The active space consists of six electrons (out of *N*) in six fragment orbitals. For the applications considered below, the active orbitals are taken to be the frontier orbitals of each fragment (HOMO and LUMO denoted as H_D_, L_D_, H_B_, L_B_, H_A_, L_A_). The use of frontier orbitals is common in SF studies.^1−3,19,23,28,30^ Our method does not depend on the particular choice of fragment-orbital basis and is not limited to two active orbitals per fragment (see Supporting Information (SI) section 5).

In the first step of the SF process, both the initial and final states are singlets, so we consider only singlet states as SF intermediates. For the active space and using the branching diagram method, we derive analytically 40 singlet spin-spatial basis states |Ψ_*n*_⟩^SA^ (SA denotes Spin-Adapted) that include single and double inter- and intrafragment excitations (SI section 1).^37−39^ We use the Slater–Condon rules^35,36^ to obtain analytical expressions for all Hamiltonian matrix elements between these states, *H*_*n*,*m*_^el^ = ^SA^⟨Ψ_*n*_|*Ĥ*^el^|Ψ_*m*_⟩^SA^ (see SI section 2).

The singly excited basis states can be categorized as locally excited (LE) and charge transfer (CT). LE states have an excited electron and a hole on the same fragment (intrafragment excitation). An example is the bridge exciton (BE) with an electron–hole (e–h) pair in the B fragment (see Scheme 1). For CT states the excited electron is on a different fragment than the hole (interfragment e–h excitation). An example is the D–A exciton (DAE) with an interfragment e–h excitation among the D and A fragments. The doubly excited (DE) states include many more excitation combinations. We denote locally doubly excited states (LDE) those that contain two intrafragment excitons (each exciton is localized within D, B, or A). In addition to locally doubly excited (LDE), there are CT doubly excited states (CTDE) that combine a CT and a LE exciton, e.g., |D^+–^B^–^A^+^⟩^SA^ (see Scheme 1). The LDE and CTDE include both correlated triplet-pair and correlated singlet-pair states (CTP and CSP, respectively).^3,15,40,41^

**Scheme 1:**
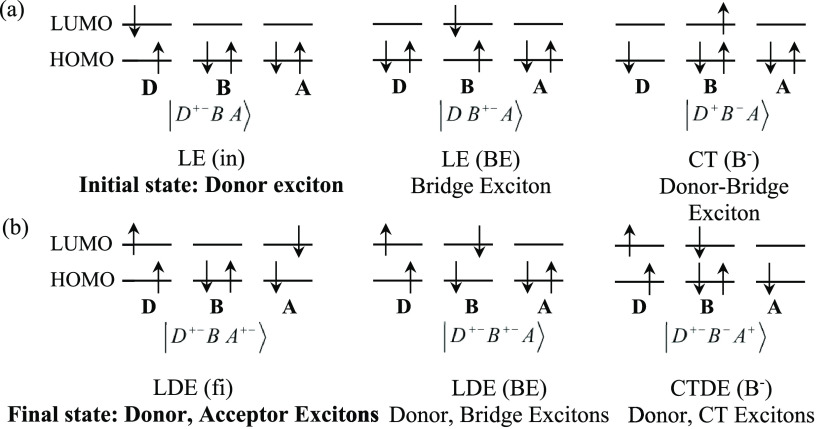
Schematic Illustration of the Notation Used to Describe the Many-Electron Basis States Relevant to the SF Pathways (a) Examples of singly excited (LE and CT) and (b) doubly excited (LDE and CTDE) states using the minimum set of orbitals per fragment (HOMO and LUMO). The kets denote Slater determinants with the spin distributions shown in the diagrams. The actual basis states used in the calculations (Table 1 and Tables S1 and S2), are spin-adapted (singlet) states that are linear combinations of Slater determinants.

In general, the basis-state energies are functions of ionization potentials, electron affinities, core terms, and Coulomb and exchange two-electron (2e) integrals, i.e.,
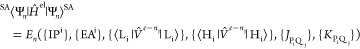
1(see SI section 2.1 for analytical expressions). The off-diagonal matrix elements are functions of one-electron (1e) Fock matrix elements, overlap matrix elements, and 2e integrals,
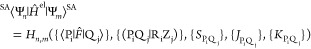
2where P, Q, R, Z = H, L and i, j = D, B, A (see SI section 2.2).

Table 1 describes the nomenclature and notation used to group the basis states. The table contains the most important groups for the discussion that follows. The first column shows the group names and the second the mathematical notation for the states. The third column gives approximate expressions for the excitation energies within each group (with respect to the DBA ground state). The approximate expressions are derived from the exact equations in SI section 2, using ab initio computations on reference systems to determine small terms that can be ignored (see below). These approximate energies are written in terms of the fragment variables *x* (*X*), *y* (*Y*), and *z* which are separately defined for the D–B fragment or A–B fragment,
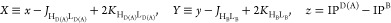
3where
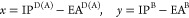
4IP denotes ionization potential and EA denotes electron affinity. The variables *X* and *Y* are the HOMO-to-LUMO exciton energies of the isolated D(A) and B fragments and are functions of *x* (*y*) and intramolecular (intrafragment) Coulomb (*J*) and exchange (*K*) integrals. We choose to write the basis-state excitation energies in terms of these variables because values for these variables are easily obtainable from experiments or computations on the fragments. The state energies in Table 1 are also functions of interfragment Coulomb and exchange integrals (denoted as “inter”).

**Table 1:** Approximate Excitation Energies of the Lowest-Lying Basis States of the D–B–A System^a^

group name	hole and electron distribution notation	approximate excitation energy
LE (in)	|D^+–^BA⟩^SA^	*X*
LDE_CTP_ (fi)	|D^+–^BA^+–^⟩_CTP_^SA^	2*X* – 2*K*_H_D_L_D__ – 2*K*_H_A_L_A__
LDE_CTP_ (BE)	|D^+–^B^+–^A⟩_CTP_^SA^, |DB^+–^A^+–^⟩_CTP_^SA^	*X* + *Y* – 2*K*_H_D(A)_L_D(A)__ – 2*K*_H_B_L_B__
LE (BE)	|DB^+–^A⟩^SA^	*Y*
CT (B^+^)	|D^–^B^+^A⟩^SA^, |DB^+^A^–^⟩^SA^	*X* – *z* + *J*_H_D(A)_L_D(A)__ – 2*K*_H_D(A)_L_D(A)__ + *J*_H_B_L_D(A)__^inter^ – 2*J*_H_B_H_D__^inter^ – 2*J*_H_B_H_A__^inter^
CT (B^–^)	|D^+^B^–^A⟩^SA^, |DB^–^A^+^⟩^SA^	*Y* + *z* + *J*_H_B_L_B__ – 2*K*_H_B_L_B__ + *J*_H_D(A)_L_B__^*inter*^ + 2*J*_H_A(D)_L_B__^*inter*^ – 2*J*_H_D(A)_H_B__^*inter*^
CT (DAE)	|D^–^BA^+^⟩^SA^, |D^+^BA^–^⟩^SA^	*X* + *J*_H_D(A)_L_D(A)__ – 2*K*_H_D(A)_L_D(A)__ + 2*J*_H_B_L_D(A)__^inter^ – 2*J*_H_A(D)_H_B__^inter^

a First column: names of the different groups of the most important singlet basis states for the D–B–A system. CTP (CSP) denotes Correlated Triplet-Pair (Correlated Singlet-Pair). Second column: mathematical notation for the spin-adapted states in each group, compare with Scheme 1. Third column: approximate excitation energies of the lowest-lying states of the D–B–A system (derived from the exact expressions in Table S3), as a function of the *X*, *Y*, and *z* parameters (see text and Scheme 2) and 2e integrals. The first two groups refer to the initial and final CTP states, denoted as (in) and (fi), respectively. In most remaining cases, the grouping is according to the B state, such as B^+^, B^–^, and the bridge excitonic (BE) state.

The excitation energies will depend on the type of solvent the D–B–A system is in. In particular, the energies of states with CT excitations are most sensitive to the solvent dielectric constant. Since we have not included an effective dielectric constant in the analytical expressions, the following analysis is more relevant to nonpolar solvents. In Scheme 2, we describe three different energetic regimes defined by the *x*, *y*, and *z* values for the D–B fragment. We label these regimes as type-I, type-II, and type-III.^42^

**Scheme 2:**
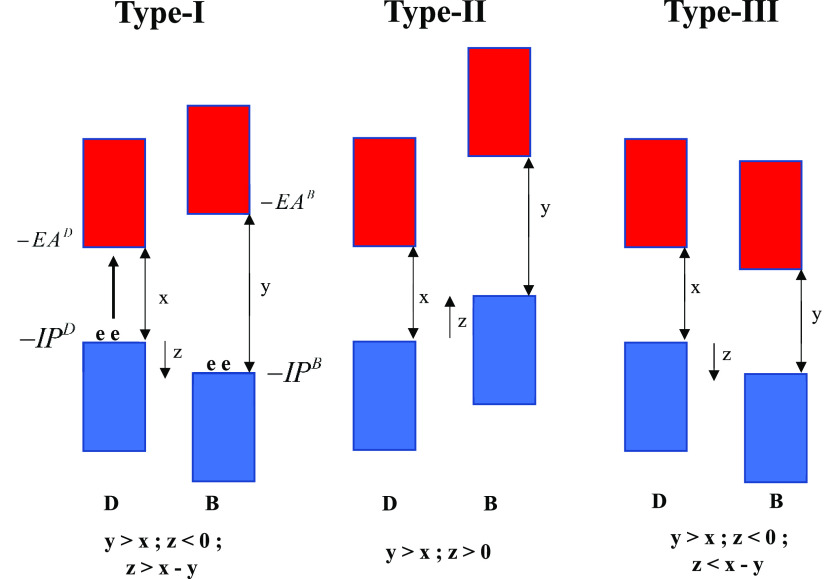
Schematic Representation of Type-I, Type-II, and Type-III D–B Regimes in the Independent-Electron Approximation Virtual orbitals are shown in red, and occupied orbitals, in blue. Since we only consider the bridge-mediated tunneling case, *y* > *x*.

In the following we consider the situation where initial photoexcitation of D–B–A creates a D-localized singlet exciton that can be approximated by |in⟩ ≈ |D^+–^BA⟩^SA^ (first row in Table 1). The coherent SF process should lead to a final state that is approximated by the D–A CTP state |fi⟩ ≈ |D^+–^BA^+–^⟩_CTP_^SA^ (second row of Table 1). All other intermediate states (third-to-final rows of Table 1 and Table S3) are off-resonant to |in⟩ and to |fi⟩ such that SF takes place by tunneling when the initial and final states come to resonance at an energy *E*_res_ (the SF rate being *k*_SF_ = |*V*_SF_|^2^ρ_FC_). The aim of our analysis is to understand how the *V*_SF_ is tuned by the identities of the D, B, and A fragments and by their relative geometries.

To make contact with realistic systems, we use some reference D–B–A groups (Figure 1) where the D and A moieties are taken to be pentacenes in a face-to-face geometry and B is pentacene (in π-stacking or non-π-stacking conformation), tetracene (in non-π-stacking conformation), or the nonconjugated 1,3-diethynyladamantyl spacer (NC1 in ref (12)). Pentacene has been studied extensively both experimentally and computationally as an individual (monomer) and as part of a larger system (dimer, trimer etc.) for the study of various SF mechanisms.^10−13,21,26^ Although the systems considered below are symmetric (D = A), the method is general and applicable to nonsymmetric systems (see SI section 5).

**Figure 1:**
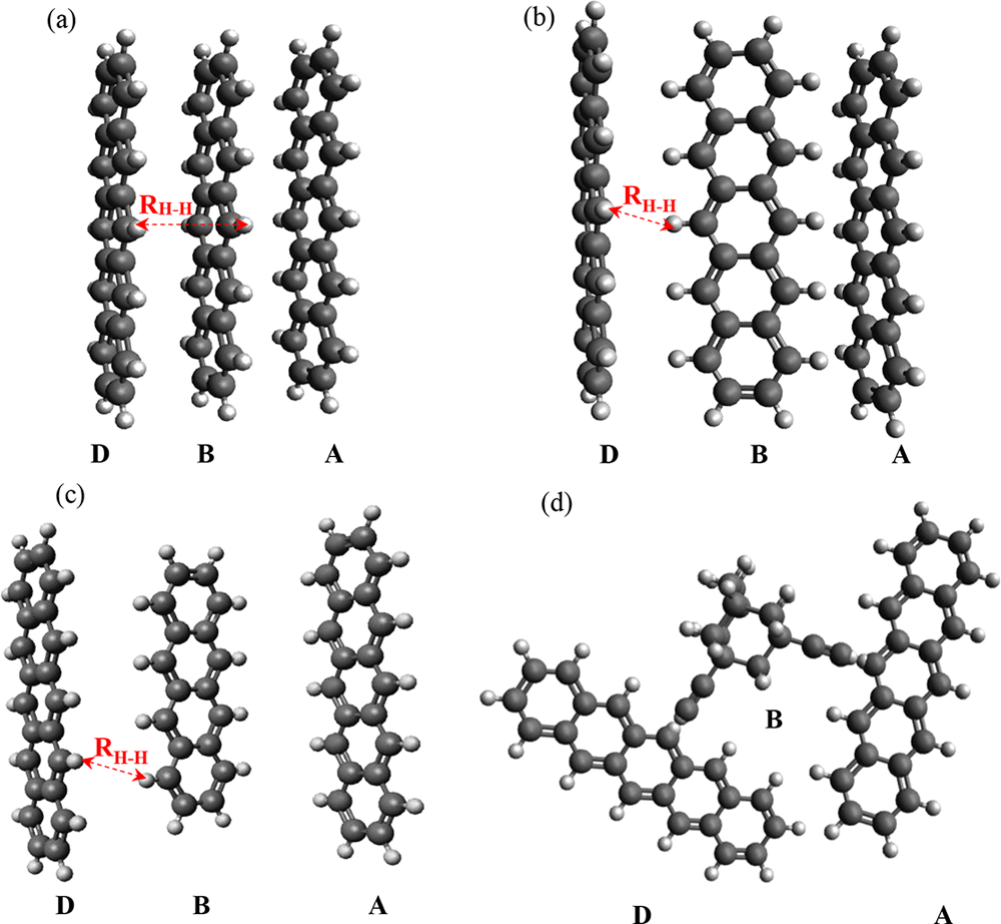
Reference D–B–A systems: (a) π-stacking pentacene trimer, (b) non-π-stacking pentacene trimer, (c) pentacene–tetracene–pentacene trimer, and (d) NC1 system of ref (12). For (a)–(c) *R*_H–H_ ≈ 3.5 Å.

For these reference systems, we use the GAMESS-US^43−45^ program in the fragment-orbital representation (6-31G(d) basis set) to compute reference values for the 1e and 2e variables in the analytical expressions of the diagonal and off-diagonal elements of the CISD Hamiltonian (eqs 1 and 2, and section S2). These variables include IPs, EAs, core terms, Fock and overlap matrix elements, and Coulomb and exchange integrals. The computed 1e and 2e variables set reference values for *x*, *y* and *z* (thus for *X*, *Y*) in eqs 3 and 4. Following this step, for each reference system, we vary *y* and *z* while keeping all other 1e and 2e parameters and *x* fixed to the reference values.

This process mimics a transformation of the B structure, with respect to the reference one, via a variation of the *IP*^B^ and EA^B^ (while keeping D and A parameters fixed). An alternative point of view is that we are varying the B-fragment exciton energy with respect to the D-fragment exciton energy (*Y* with respect to *X*). We explore how such transformations alter *V*_SF_ and the SF pathways for the fully coupled D–B–A system in the tunneling regime. *V*_SF_ is computed by exact diagonalization of the full Hamiltonian (40 states, with the exact matrix elements), setting both initial- and final-state energies equal to the resonance (tunneling) energy *E*_res_ = (*E*_in_ + *E*_fi_)/2 (Figure 3 and SI section 3). Pathway contributions to *V*_SF_ are obtained by Green’s function methods and by deleting intermediate states in the Hamiltonian and computing the effect on *V*_SF_ (SI section 3).^46−49^ Thus the *V*_SF_ plots in Figure 2 are exact, involving diagonalization, whereas the dominant pathway structures in Figure 3 are approximate interpretations of the exact results.

**Figure 2:**
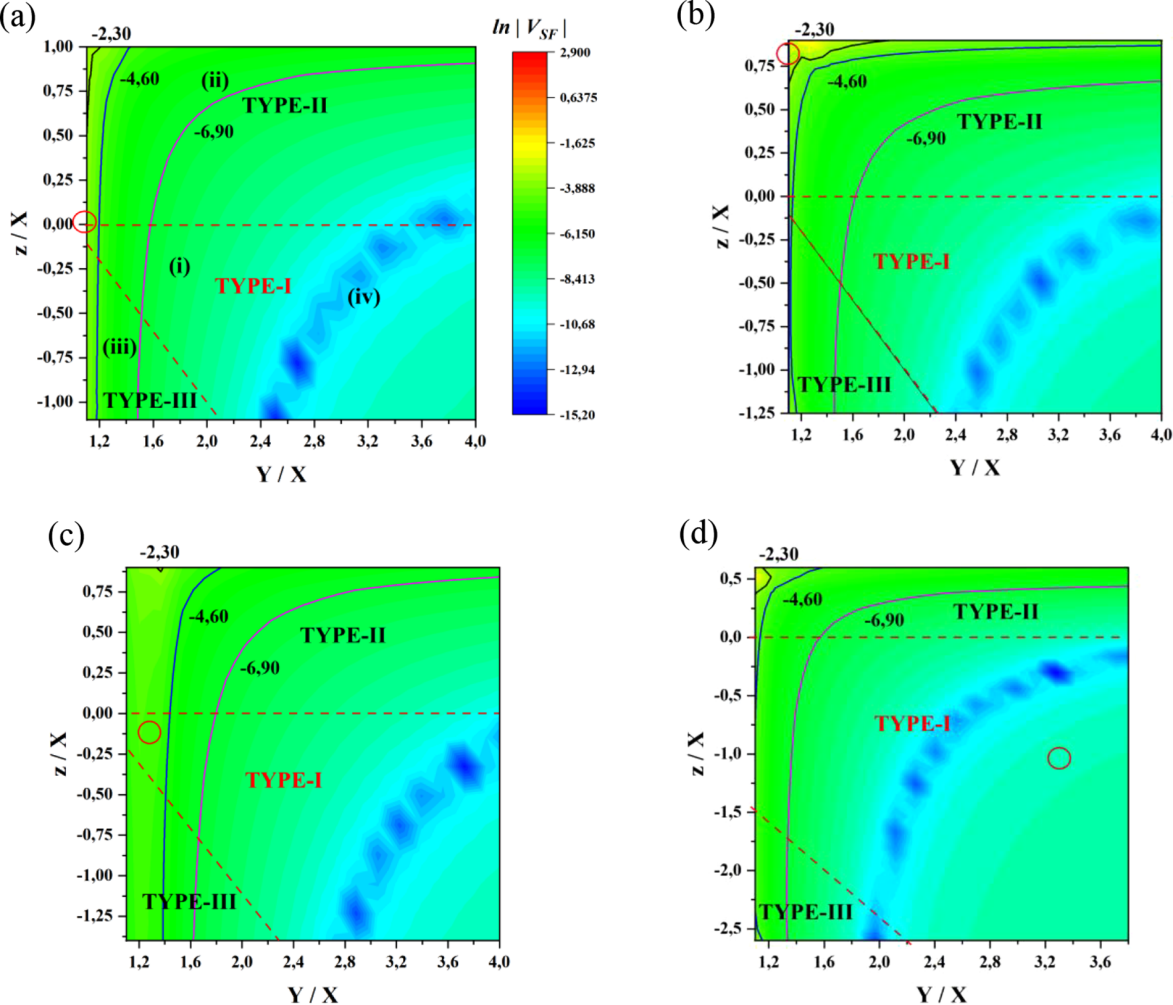
ln |*V*_SF_| plot as a function of *Y*/*X* and *z*/*X* parameters for (a) π-stacking conformation shown in Figure 1a, (b) the non-π-stacking conformation shown in Figure 1b, (c) the pentacene–tetracene–pentacene molecular system of Figure 1c, and (d) the NC1 molecular system of Figure 1d. The dashed lines outline the three regimes defined in Scheme 2. The black contour corresponds to a coupling |*V*_SF_| = 10^–1^ eV, the blue to |*V*_SF_| = 10^–2^ eV, and the magenta lines to |*V*_SF_| = 10^–3^ eV. The colormap scaling is the same for all plots. The circles represent the *Y*/*X*, *z*/*X*, and *V*_SF_ values of the reference systems in Figure 1. The labels (i)–(iv) refer to the approximate pathway structures discussed in the text.

**Figure 3:**
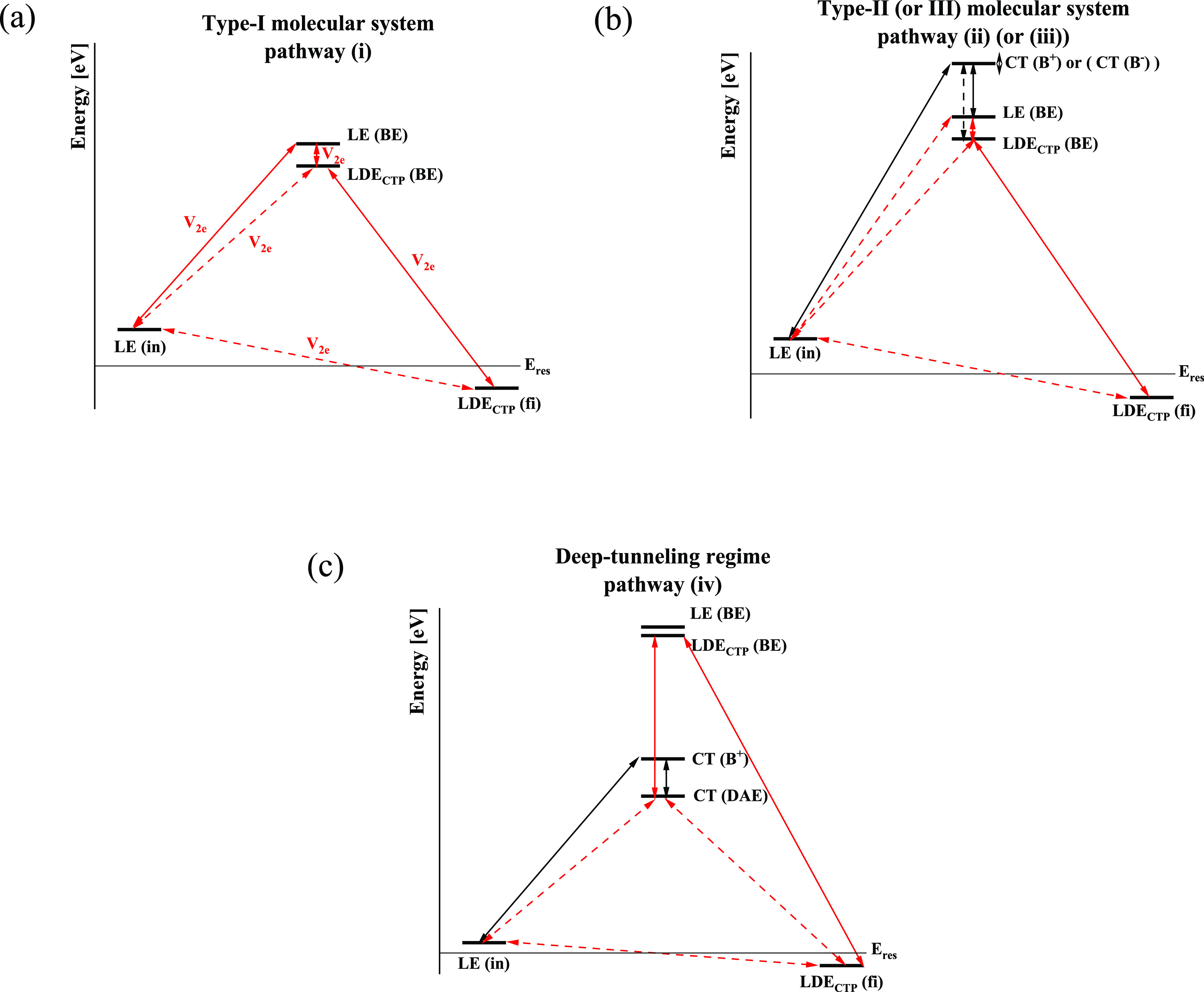
Schematic representation of the energies and the coupling structure of the intermediate states of Table 1, for (a) the type-I and (b) the type-II (or -III) energetic regime and (c) the deep-tunneling case with *Y*/*X* ≫ 1. *V*_2e_ (red arrows) denotes coupling dominated by 2e matrix elements, *V*_2e_ = ⟨Ψ_*n*_|*V̂*^2e^|Ψ_*m*_⟩, and *V*_1e_ (black arrows) denotes coupling dominated by 1e matrix elements, *V*_1e_ = ⟨Ψ_*n*_|*ĥ*^1e^|Ψ_*m*_⟩. The weaker matrix elements are represented by dotted lines and *E*_res_ = (*E*_in_ + *E*_fi_)/2. These dominant pathway structures relate to symmetric D–B–A systems for which D = A and to nonpolar solvents.

Figure 2 shows contour plots of ln |*V*_SF_(*Y*/*X*, *z*/*X*)| for the reference systems of Figure 1, where *X* is fixed to the D(A) pentacene value. Figure 2 panels a–d relate to the reference systems 1a, 1b, 1c, and 1d, respectively. The circles in the plots correspond to the coupling values for the computed *Y* and *z* of the reference systems. The labels (i), (ii), (iii), and (iv) refer to the pathway structures contributing to the SF couplings (see following discussion).

The above contour plots for the SF coupling variation relating to the different system types (and to the different reference systems) have similar generic features. |*V*_SF_| increases as *Y* → *X* (*Y* > *X*), i.e., as quasi-resonance between the initial (final) state and the BE states is approached from off-resonance (quasi-resonance corresponds to the leftmost side of contour plots). The maximum |*V*_SF_| value for all types (I–III) is |*V*_SF_| ∼ 10^–1^ eV (ln |*V*_SF_| ∼ −2.3). For the type-I case the maximum coupling is only due to the BE-character states because these are the closest in energy to the initial and final states. The dominant type-I pathway structure is (i)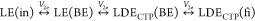 and it is mediated by purely 2e interactions, i.e., *V*_2e_ = ⟨Ψ_*n*_|*V̂*^2e^|Ψ_*m*_⟩. In contrast, the maximum coupling region for type-II or type-III involves CT (B^+^) or (B^–^) excitons, respectively, in addition to the above-mentioned BE states. These D(A)–B CT states have lower energies as compared to the type-I case and approach the energies of the BE-character states. Thus, the dominant pathway structure for type-II is (ii)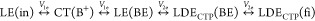 and for type-III is (iii)
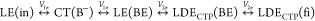
Both pathways involve 2e and 1e interactions (*V*_1e_ = ⟨Ψ_*n*_|*ĥ*^1e^|Ψ_*m*_⟩). These pathway structures relate to symmetric D–B–A systems (D = A) and to nonpolar solvents. Polar solvents would lower CT-state energies.

The three pathway structures are largely preserved as *Y* increases with respect to *X*, i.e., moving from the quasi-resonant to the off-resonant regime (from the left to the right side of the contour plots). The *Y*/*X* increase implies an increase in the energies of the BE-character states, thus a weakening of the |*V*_SF_| magnitude for all the system types. In the deep-tunneling regime (*Y*/*X* ≫ 1, rightmost side of contour plots), the relative contribution from the high-energy BE-character states is reduced and D–A CT excitons (DAE) become important. For example, for the NC1 reference structure, |*V*_SF_| ∼ 10^–4^ eV (red circle in type-I region of Figure 2d). This value is consistent with the results from Basel et al.^12^ who studied this molecular system using CASSCF calculations on a 4e4o active space with D(A)-centered orbitals (XMCQDPT/DZV level of theory). Our computations, which also include bridge-centered states, show that the dominant pathway structure is (iv)
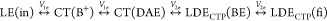
D–A CT excitons have the lowest energies and are major contributors to |*V*_SF_|. The lowest-coupling blue region in the deep-tunneling regime arises from destructive pathway interferences.

Below, we show schematic representations of the most important states (energies) and the corresponding interstate coupling topology for the different types of systems discussed above. We find that the CT doubly excited states (CTDE in Scheme 1) and all the CSP states (included in the full-Hamiltonian computations) do not contribute significantly to *V*_SF_ due to their high energies.

In summary, for each system type considered here and for symmetric D–B–A systems, we identified the dominant bridge-mediated SF pathways and the corresponding SF intermediates (Figure 3). We find that all system types can give similar magnitudes for the SF coupling even though the underlying pathway structures differ. An important conclusion is that for all system types the bridge can enhance *V*_SF_ through the CTP states |D^+–^B^+–^A⟩_CTP_ (|DB^+–^A^+–^⟩_CTP_) and the single-exciton state |DB^+–^A⟩. For type-II and type-III systems, D(A)–B CT states |D^–^B^+^A⟩ (|DB^+^A^–^⟩) or |D^+^B^–^A⟩ (|DB^–^A^+^⟩) also contribute significantly to *V*_SF_. Explicit expressions for all these intermediate states are given in the SI section 1. We also find that the differences between the ln |*V*_SF_| contour plots in Figure 2 are mainly due to differences in intermediate-state energies rather than the interstate couplings *V*_1e_ and *V*_2e_ shown in Figure 3 (see SI section 4). However, molecular motions and disorder could modulate the interstate couplings such that the pathways contributing to the ensemble-averaged  may show some differences as compared to the static *V*_SF_.

Nevertheless, the bridge-tuning of *V*_SF_ is most sensitive to energy differences between the above-mentioned intermediate states and the initial |in⟩ = |D^+–^BA⟩ (given that the final state |D^+–^BA^+–^⟩_CTP_ has lower energy than the initial). We have shown analytically that these energy differences, *ΔE*_Ψ_ = *E*_|Ψ⟩_ – *E*_|in⟩_, can be approximated in terms of D(A) and B fragment exciton energies *X* and *Y*, a few intrafragment and interfragment Coulomb and exchange integrals and differences in fragment IPs, *z* = IP^B^ – IP^D^ (Table 1). These variables are easily computed using fragment calculations. Some of them, such as IPs and fragment singlet-exciton energies (*X* and *Y*), can be approximated from experiments on the fragments.

Figure 3 panels a and b show that the biexcitonic CTP states |D^+–^B^+–^A⟩_CTP_ (|DB^+–^A^+–^⟩_CTP_), whose equations are given in Table S2, are “bottleneck” states for *V*_SF_ for all system types, in the sense that they are the intermediates that are always strongly coupled to the fission product |D^+–^BA^+–^⟩_CTP_. Therefore, an approximate approach to bridge-tuning of *V*_SF_ is to modulate the energies of the “bottleneck” intermediates and their couplings to the final SF state. From Table 1 we conclude that rough estimates of these energies (for the systems under study in the off-resonant regime) are given by the sums of the energies of the D(A) and B fragment triplet excitons (exact energies are shown in Table S3),
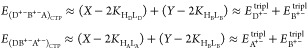
5

The couplings to the final SF state are simple exchange integrals that are easy to compute,
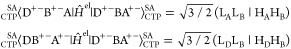
6(we find maximum values of 0.1 eV). The “bottleneck” intermediates are accessed from the initial |D^+–^BA⟩^SA^ state through different pathways, depending on the system type. For type-I, they are mainly accessed through the LE (BE) state |DB^+–^A⟩^SA^ via 2e interactions that involve pairs of exchange integrals. For type-II and -III, the “bottleneck” states are mainly accessed from the initial state via BE and D–B CT states (II, |D^–^B^+^A⟩^SA^; III, |D^+^B^–^A⟩^SA^) with pathways that involve both 1e and 2e interactions (see SI section 2).

The concept of “bottleneck” states sheds light on the bridge-resonance effect in SF. The systems studied in refs (25) and (26) are type-I (Figure 3a). The D(A) and B HOMO−LUMO gaps in our notation are *x* and *y*. Lowering the B HOMO–LUMO gap corresponds to *y* → *x* (*Y*/*X* → 1), leading to an enhancement of the SF coupling (moving toward the leftmost side of the contour plots in Figure 2). As *y* → *x*, the “bottleneck”-state energy is reduced due to lowering of the bridge triplet-exciton energy (eq 5 and Table 1).

In conclusion, the semianalytical approach is a useful tool to derive, interpret, and predict structure–function relationships and electronic pathways for bridge-mediated SF rates. It can also be used to guide searches of candidate D–B–A systems given target SF coupling magnitudes. These candidate systems may then be studied at a higher level of quantum chemical theory and tested by experiment.

## Supporting Information Available

Derivation of the many-electron spin-adapted basis set using the branching diagram method in the context of the CISD method and D, B, A HOMO/LUMO orbitals and tables of the singly- and doubly-excited spin-adapted states; derivation of the many-electron DBA Hamiltonian matrix elements using the above basis set and tables of analytical expressions; description of the SF effective coupling computation using exact diagonalization of the Hamiltonian and SF coupling pathways; analysis of the relative importance of intermediate-state energies as compared to interstate couplings in determining the SF effective coupling and a figure of the difference contour plots; discussion of the generality of the method (non-frontier-orbital basis, nonsymmetric D–B–A systems, fragment excitations that involve more than one pair of electron and hole orbitals) (PDF)
Transparent Peer Review report available (PDF)

## References

[ref1] Smith M. B.; Michl J. Singlet Fission. Chem. Rev. 2010, 110, 6891–6936. 10.1021/cr1002613. 21053979

[ref2] Smith M. B.; Michl J. Recent Advances in Singlet Fission. Annu. Rev. Phys. Chem. 2013, 64, 361–386. 10.1146/annurev-physchem-040412-110130. 23298243

[ref3] Casanova D. Theoretical Modeling of Singlet Fission. Chem. Rev. 2018, 118, 7164–7207. 10.1021/acs.chemrev.7b00601. 29648797

[ref4] Japahuge A.; Zeng T. Theoretical Studies of Singlet Fission: Searching for Materials and Exploring Mechanisms. ChemPlusChem. 2018, 83, 146–182. 10.1002/cplu.201700489. 31957288

[ref5] Hanna M. C.; Nozik A. J. Solar conversion efficiency of photovoltaic and photoelectrolysis cells with carrier multiplication absorbers. J. Appl. Phys. 2006, 100, 074510 10.1063/1.2356795.

[ref6] Burdett J. J.; Bardeen C. J. The Dynamics of Singlet Fission in Crystalline Tetracene and Covalent Analogs. Acc. Chem. Res. 2013, 46 (6), 1312–1320. 10.1021/ar300191w. 23360099

[ref7] Pensack R. D.; Tilley A. J.; Parkin S. R.; Lee T. S.; Payne M. M.; Gao D.; Jahnke A. A.; Oblinsky D. G.; Li P.-F.; Anthony J. E.; et al. Exciton Delocalization Drives Rapid Singlet Fission in Nanoparticles of Acene Derivatives. J. Am. Chem. Soc. 2015, 137 (21), 6790–6803. 10.1021/ja512668r. 25946670

[ref8] Burdett J. J.; Bardeen C. J. Quantum Beats in Crystalline Tetracene Delayed Fluorescence Due to Triplet Pair Coherences Produced by Direct Singlet Fission. J. Am. Chem. Soc. 2012, 134, 8597–8607. 10.1021/ja301683w. 22530591

[ref9] Zeng T.; Hoffmann R.; Ananth N. The Low-Lying Electronic States and Their Roles in Singlet Fission. J. Am. Chem. Soc. 2014, 136, 5755–5764. 10.1021/ja500887a. 24697685

[ref10] Zimmerman P. M.; Zhang Z.; Musgrave C. B. Singlet Fission in pentacene through multi-exciton quantum states. Nat. Chem. 2010, 2, 648–652. 10.1038/nchem.694. 20651727

[ref11] Basel B. S.; Zirzlmeier J.; Hetzer C.; Reddy S. R.; Phelan B. T.; Krzyaniak M. D.; Volland M. K.; Coto P. B.; Young R. M.; Clark T.; et al. Evidence for Charge-Transfer Mediation in the Primary Events of Singlet Fission in a Weakly Coupled Pentacene Dimer. Chem. 2018, 4 (5), 1092–1111. 10.1016/j.chempr.2018.04.006.

[ref12] Basel B. S.; Zirzlmeier J.; Hetzer C.; Phelan B. T.; Krzyaniak M. D.; Reddy S. R.; Coto P. B.; Horwitz N. E.; Young R. M.; White F. J.; et al. Unified model for singlet fission within a non-conjugated covalent pentacene dimer. Nat. Commun. 2017, 8, 15171 10.1038/ncomms15171. 28516916 PMC5493541

[ref13] Papadopoulos I.; Zirzlmeier J.; Hetzer C.; Bae Y. J.; Krzyaniak M. D.; Wasielewski M. R.; Clark T.; Tykwinski R. R.; Guldi D. M. Varying the Interpentacene Electronic Coupling to Tune Singlet Fission. J. Am. Chem. Soc. 2019, 141, 6191–6203. 10.1021/jacs.8b09510. 30854854

[ref14] Abraham V.; Mayhall N. J. Revealing the contest between triplet-triplet exchange and triplet-triplet energy transfer coupling in correlated triplet pair states in singlet fission. J. Phys. Chem. Lett. 2021, 12, 10505–10514. 10.1021/acs.jpclett.1c03217. 34677988

[ref15] Scholes G. D. Correlated Pair States Formed by Singlet Fission and Exciton-Exciton Annihilation. J. Phys. Chem. A 2015, 119, 12699–12705. 10.1021/acs.jpca.5b09725. 26595530

[ref16] Pensack R. D.; Ostroumov E. E.; Tilley A. J.; Mazza S.; Grieco C.; Thorley K. J.; Asbury J. B.; Seferos D. S.; Anthony J. E.; Scholes G. D. Observation of Two Triplet-Pair Intermediates in Singlet Exciton Fission. J. Phys. Chem. Lett. 2016, 7, 2370–2375. 10.1021/acs.jpclett.6b00947. 27281713

[ref17] Taffet E. J.; Beljonne D.; Scholes G. D. Overlap-Driven Splitting of Triplet Pairs in Singlet Fission. J. Am. Chem. Soc. 2020, 142, 20040–20047. 10.1021/jacs.0c09276. 33190497

[ref18] Li X.; Parrish R. M.; Liu F.; Schumacher S. I. L. K.; Martínez T. J. An Ab Initio Exciton Model Including Charge-Transfer Excited States. J. Chem. Theory Comput. 2017, 13, 3493–3504. 10.1021/acs.jctc.7b00171. 28617595

[ref19] Mirjani F.; Renaud N.; Gorczak N.; Grozema F. C. Theoretical Investigation of Singlet Fission in Molecular Dimers: The Role of Charge Transfer States and Quantum Interference. J. Phys. Chem. C 2014, 118, 14192–14199. 10.1021/jp503398a.

[ref20] Korovina N. V.; Das S.; Nett Z.; Feng X.; Joy J.; Haiges R.; Krylov A. I.; Bradforth S. E.; Thompson M. E. Singlet Fission in a Covalently Linked Cofacial Alkynyltetracene Dimer. J. Am. Chem. Soc. 2016, 138, 617–627. 10.1021/jacs.5b10550. 26693957

[ref21] Zirzlmeier J.; Casillas R.; Reddy S. R.; Coto P. B.; Lehnherr D.; Chernick E. T.; Papadopoulos I.; Thoss M.; Tykwinski R. R.; Guldi D. M. Solution-based intramolecular singlet fission in cross-conjugated pentacene dimers. Nanoscale. 2016, 8, 10113–10123. 10.1039/C6NR02493A. 27122097

[ref22] Ito S.; Nagami T.; Nakano M. Design Principles of Electronic Couplings for Intramolecular Singlet Fission in Covalently-Linked Systems. J. Phys. Chem. A 2016, 120, 6236–6241. 10.1021/acs.jpca.6b07153. 27448100

[ref23] Ito S.; Nagami T.; Nakano M. Molecular design for efficient singlet fission. J. Photochem. Photobiol. 2018, 34, 85–120. 10.1016/j.jphotochemrev.2018.01.002.

[ref24] Kumarasamy E.; Sanders S. N.; Tayebjee M. J. Y.; Asadpoordarvish A.; Hele T. J. H.; Fuemmeler E. G.; Pun A. B.; Yablon L. M.; Low J. Z.; Paley D. W.; et al. Tuning Singlet Fission in π-Bridge-π Chromophores. J. Am. Chem. Soc. 2017, 139, 12488–12494. 10.1021/jacs.7b05204. 28799752

[ref25] Korovina N. V.; Joy J.; Feng X.; Feltenberger C.; Krylov A. I.; Bradforth S. E.; Thompson M. E. Linker-Dependent Singlet Fission in Tetracene Dimers. J. Am. Chem. Soc. 2018, 140, 10179–10190. 10.1021/jacs.8b04401. 30016102

[ref26] Parenti K. R.; He G.; Sanders S. N.; Pun A. B.; Kumarasamy E.; Sfeir M. Y.; Campos L. M. Bridge Resonance Effects in Singlet Fission. J. Phys. Chem. A 2020, 124, 9392–9399. 10.1021/acs.jpca.0c08427. 33138366

[ref27] Rais D.; Toman P.; Pfleger J.; Acharya U.; Panthi Y. R.; Menšík M.; Zhigunov A.; Thottappali M. A.; Vala M.; Marková A.; et al. Singlet Fission in Thin Solid Films of Bis(thienyl) diketopyrrolopyrroles. ChemPlusChem. 2020, 85, 2689–2703. 10.1002/cplu.202000623. 33332757

[ref28] Buchanan E. A.; Havlas Z.; Michl J. Optimal Arrangements of Tetracene Molecule Pairs for Fast Singlet Fission. Bull. Chem. Soc. Jpn. 2019, 92, 1960–1971. 10.1246/bcsj.20190229. 31463501

[ref29] Zaykov A.; Felkel P.; Buchanan E. A.; Jovanovic M.; Havenith R. W. A.; Kathir R. K.; Broer R.; Havlas Z.; Michl J. Singlet Fission Rate: Optimized Packing of a Molecular Pair. Ethylene as a Model. J. Am. Chem. Soc. 2019, 141, 17729–17743. 10.1021/jacs.9b08173. 31509712

[ref30] Buchanan E. A.; Havlas Z.; Michl J. Singlet Fission: Optimization of Chromophore Dimer Geometry. Adv. Quantum Chem. 2017, 75, 175–227. 10.1016/bs.aiq.2017.03.005.

[ref31] Havlas Z.; Michl J. Guidance for Mutual Disposition of Chromophores for Singlet Fission. Isr. J. Chem. 2016, 56, 96–106. 10.1002/ijch.201500054.

[ref32] Sandoval-Salinas M. E.; Casanova D. The Doubly Excited State in Singlet Fission. ChemPhotoChem. 2021, 5, 282–293. 10.1002/cptc.202000211.

[ref33] Casanova D.; Krylov A. I. Quantifying local exciton, charge resonance, and multiexciton character in correlated wave functions of multichromophoric systems. J. Chem. Phys. 2016, 144, 014102 10.1063/1.4939222. 26747796

[ref34] Luzanov A. V.; Casanova D.; Feng X.; Krylov A. I. Quantifying charge resonance and multiexciton character in coupled chromophores by charge and spin cumulant analysis. J. Chem. Phys. 2015, 142, 224104 10.1063/1.4921635. 26071698

[ref35] Szabo A.; Ostlund N. S. Modern Quantum Chemistry: Introduction to Advanced Electronic Structure Theory; Dover: Mineola, NY, 1996.

[ref36] Sherrill C. D. Derivation of the Configuration Interaction Singles (CIS) Method for Various Single Determinant References and Extensions to Include Selected Double Substitutions (XCIS). Sch. Chem. Biochem. 1996, 1–14.

[ref37] Pauncz R. The Construction of Spin Eigenfunctions: An Exercise Book, 1st ed.; Springer: Boston, MA, 2000.

[ref38] Pauncz R. The Symmetric Group in Quantum Chemistry; CRC Press: Boca Raton, FL, 1995.

[ref39] Grabenstetter J. E.; Tseng T. J.; Grein F. Generation of genealogical spin eigenfunctions. Int. J. Quantum Chem. 1976, 10 (1), 143–149. 10.1002/qua.560100112.

[ref40] Miyata K.; Conrad-Burton F. S.; Geyer F. L.; Zhu X.-Y. Triplet Pair States in Singlet Fission. Chem. Rev. 2019, 119, 4261–4292. 10.1021/acs.chemrev.8b00572. 30721032

[ref41] Breen I.; Tempelaar R.; Bizimana L. A.; Kloss B.; Reichman D. R.; Turner D. B. Triplet Separation Drives Singlet Fission after Femtosecond Correlated Triplet Pair Production in Rubrene. J. Am. Chem. Soc. 2017, 139, 11745–11751. 10.1021/jacs.7b02621. 28763611

[ref42] Wang S.; Tian H.; Ren C.; Yu J.; Sun M. Electronic and optical properties of heterostructures based on transition metal dichalcogenides and graphene-like zinc oxide. Sci. Rep. 2018, 8, 12009 10.1038/s41598-018-30614-3. 30104708 PMC6089903

[ref43] Dupuis M.; Spangler D.; Wendolowski J. J. National Resource for Computations in Chemistry Software Catalog; University of California, Berkeley, CA, Program QG01, 1980.

[ref44] Schmidt M. W.; Baldridge K. K.; Boatz J. A.; Elbert S. T.; Gordon M. S.; Jensen J. H.; Koseki S.; Matsunaga N.; Nguyen K. A.; Su S.; et al. General Atomic and Molecular Electronic Structure System. J. Comput. Chem. 1993, 14 (11), 1347–1363. 10.1002/jcc.540141112.

[ref45] Advances in electronic structure theory: GAMESS a decade later. In Theory and Applications of Computational Chemistry: the first forty years; Gordon M. S., Schmidt M. W., Dykstra C. E., Frenking G., Kim K. S., Scuseria G. E., Eds.; Elsevier: Amsterdam, 2005; pp 1167–1189.

[ref46] Löwdin P.-O. Studies in Perturbation Theory. IV. Solution of Eigenvalue Problem by Projection Operator Formalism. J. Math. Phys. 1962, 3 (5), 969–982. 10.1063/1.1724312.

[ref47] Skourtis S. S.; Beratan D. N.; Onuchic J. N. The two-state reduction for electron and hole transfer in bridge-mediated electron-transfer reactions. Chem. Phys. 1993, 176, 501–520. 10.1016/0301-0104(93)80258-B.

[ref48] Skourtis S. S.; Beratan D. N. Theories of Structure-Function Relationships for Bridge-Mediated Electron Transfer Reactions. Adv. Chem. Phys. 2007, 106, 377–452. 10.1002/9780470141656.ch8.

[ref49] Castellanos M. A.; Huo P. Enhancing Singlet Fission Dynamics by Suppressing Destructive Interference between Charge-Transfer Pathways. J. Phys. Chem. Lett. 2017, 8, 2480–2488. 10.1021/acs.jpclett.7b00972. 28520444

