# Supplementary material for: The Role of Bridge-State Intermediates in Singlet Fission for Donor–Bridge–Acceptor Systems: A Semianalytical Approach to Bridge-Tuning of the Donor–Acceptor Fission Coupling
Source: J Phys Chem Lett. 2022 Jan 20;13(3):939–46. doi: 10.1021/acs.jpclett.1c03700 (PMC9836358; doi:10.1021/acs.jpclett.1c03700)
Supplement: Supplementary file 1 — jz1c03700_si_001.pdf [file jz1c03700_si_001.pdf]

Supporting Information

The Role of Bridge-State Intermediates in Singlet Fission for Donor-Bridge-Acceptor Systems: A Semianalytical Approach to Bridge-Tuning of the Donor-Acceptor Fission Coupling

Stephanie Valianti[1] and Spiros S. Skourtis*[1]

[1]Department of Physics, University of Cyprus, 1678, Nicosia, Cyprus

*Email: skourtis@ucy.ac.cy

1. Many-Electron Spin-Adapted Basis Set

In the first step of the SF process both the initial and final states are singlets, so we consider only singlet states as intermediates for this step (within the CISD formalism, these include single and double excitations). The many-electron states we use describe the D, B or A localization of the excited electron (e) and the hole (h), and are eigenstates of the total spin. They can be represented by linear combinations of singly- and doubly excited $N-$ electron determinants.

The spin eigenfunctions are constructed via the branching diagram method (using the Yamanouchi-Kotani functions).[1-3] For N $s=1/2$ spins we construct eigenfunctions of $\hat{S}_N^2$, $\hat{S}_{z,N}$ (with quantum numbers S_N and M_S, respectively). For a given quantum number S_N there are different groups of such eigenfunctions, each group containing $2S_N+1$ $\hat{S}_{z,N}$ eigenfunctions. Given a value of S_N, the number of such groups for N spins is $f(N,S_N)=\begin{pmatrix} N \\ \frac{1}{2}N-S_N \end{pmatrix}-\begin{pmatrix} N \\ \frac{1}{2}N-S_N-1 \end{pmatrix}$. We denote the eigenfunctions of $\hat{S}_N^2$ and $\hat{S}_{z,N}$ belonging to the kth group by $X(k)\equiv X(N,S_N,M_S\,;k)$. The construction of the eigenfunctions in each group is done iteratively. Given the $X\left(N-1,S_{N-1},M_{S_{N-1}}\,;k\right)$ eigenfunctions we obtain new eigenfunctions corresponding to either $S_N=S_{N-1}+1/2$ or $S_N=S_{N-1}-1/2$ by adding or subtracting the spin of the Nth electron, respectively.[1,2]

The spin-spatial state with a prespecified occupation of spatial orbitals, is constructed by first multiplying a $X(k)$ by a product of the prespecified spatial orbitals, $\Psi(A,B,...,\Omega)=\psi_A(1)\,\psi_B(2)...\psi_\Omega(N)$. The $\psi's$ are one-electron (1e) orbitals and

$A, B, ..., \Omega$ are the orbital labels (e.g., H_D for the HOMO of the Donor fragment, L_A for the LUMO of Acceptor fragment, etc.). Thus, the spin-spatial antisymmetric wavefunctions can be written in a multi-determinant form as $\left|\Psi(A,B,...,\Omega); X(k)\right\rangle^{SA} = \hat{A}_N \Psi(A,B,...,\Omega) X(k)$ where $\hat{A}_N$ is the antisymmetrization operator. [4,5] $\left|...\right\rangle^{SA}$ denotes a "Spin-Adapted" state that most often involves a linear combination of Slater determinants of 1e spin orbitals (denoted simply by $\left|...\right\rangle$).

Since we consider only singlet states, we will not use a total-spin label in our notation. For our active space we can create $f(N=4, S_N=0)=2$ groups of spin eigenfunctions of $\hat{S}_N^2$ that describe the possible states of the system. Below in Table S1, are shown some examples of the most important singly excited states (LE and CT) used in SF pathways. Some of these states are illustrated in Scheme 1 of the main text. $\left|\Psi_a^r\right\rangle$ denotes a singly excited determinant in which an electron, which occupied spin-orbital χ_a in the Hartree-Fock (HF) ground state of the D-B-A $N-$ electron system $\left|\Psi_{0,N}\right\rangle$, has been promoted to a virtual spin-orbital χ_r. In such determinants, χ_a (or a) is used to denote spin-orbital with spin up and $\bar{\chi}_a$ (or $\bar{a}$) spin-orbital with spin down. [4,6] The superscript "+" ("-") denotes hole (electron).

Table S1. The Most Important Singly Excited Spin-Adapted (SA) Singlet States Used as a Basis to Represent the D-B-A Many-Electron Hamiltonian [α]

h-e distribution notation	Expression
$\left\|D^{+-}BA\right\rangle^{SA}$	$\frac{1}{\sqrt{2}}\left(\left\|\Psi_{H_D}^{L_D}\right\rangle+\left\|\Psi_{\bar{H}_D}^{\bar{L}_D}\right\rangle\right)$
$\left\|DB^{+-}A\right\rangle^{SA}$	$\frac{1}{\sqrt{2}}\left(\left\|\Psi_{H_B}^{L_B}\right\rangle+\left\|\Psi_{\bar{H}_B}^{\bar{L}_B}\right\rangle\right)$
$\left\|D^{+}B^{-}A\right\rangle^{SA}$	$\frac{1}{\sqrt{2}}\left(\left\|\Psi_{H_D}^{L_B}\right\rangle+\left\|\Psi_{\bar{H}_D}^{\bar{L}_B}\right\rangle\right)$
$\left\|D^{-}B^{+}A\right\rangle^{SA}$	$\frac{1}{\sqrt{2}}\left(\left\|\Psi_{H_B}^{L_D}\right\rangle+\left\|\Psi_{\bar{H}_B}^{\bar{L}_D}\right\rangle\right)$
$\left\|D^{+}BA^{-}\right\rangle^{SA}$	$\frac{1}{\sqrt{2}}\left(\left\|\Psi_{H_D}^{L_A}\right\rangle+\left\|\Psi_{\bar{H}_D}^{\bar{L}_A}\right\rangle\right)$

[α] First column: mathematical notation for the spin-adapted many-electron basis states. Second column: spin-spatial multi-electronic states as linear combinations of singly excited Slater determinants.

The doubly excited (DE) states include many more excitation combinations. In these cases, using the branching diagram method, we can construct correlated triplet-pair DE states (denoted by CTP) and correlated singlet-pair DE states (denoted by CSP). Below in Table S2, we give some examples of CTP and CSP doubly excited states (LDE and CTDE). [5,7-9] $\left|\Psi_{a,b}^{r,p}\right\rangle$ denotes doubly excited determinants.

Table S2. Examples of Doubly Excited Spin-Adapted (SA) States Used as a Basis to Represent the D-B-A Hamiltonian [α]

**h-e distribution notation**	**Expression**
$\left\|D^{+-}BA^{+-}\right\rangle^{SA}_{CTP}$	$\sqrt{\frac{1}{3}}\left(\left\|\Psi^{\bar{L}_A L_D}_{\bar{H}_D H_A}\right\rangle+\left\|\Psi^{L_A \bar{L}_D}_{H_D \bar{H}_A}\right\rangle-\frac{1}{2}\begin{pmatrix}\left\|\Psi^{\bar{L}_D \bar{L}_A}_{\bar{H}_D \bar{H}_A}\right\rangle-\left\|\Psi^{L_D \bar{L}_A}_{H_D \bar{H}_A}\right\rangle\\-\left\|\Psi^{\bar{L}_D L_A}_{\bar{H}_D H_A}\right\rangle+\left\|\Psi^{L_D L_A}_{H_D H_A}\right\rangle\end{pmatrix}\right)$
$\left\|D^{+-}BA^{+-}\right\rangle_{CSP}$	$\frac{1}{2}\left(\left\|\Psi^{\bar{L}_D \bar{L}_A}_{\bar{H}_D \bar{H}_A}\right\rangle+\left\|\Psi^{L_D \bar{L}_A}_{H_D \bar{H}_A}\right\rangle+\left\|\Psi^{\bar{L}_D L_A}_{\bar{H}_D H_A}\right\rangle+\left\|\Psi^{L_D L_A}_{H_D H_A}\right\rangle\right)$
$\left\|D^{+-}B^{+-}A\right\rangle^{SA}_{CTP}$	$\sqrt{\frac{1}{3}}\left(\left\|\Psi^{\bar{L}_B L_D}_{\bar{H}_D H_B}\right\rangle+\left\|\Psi^{L_B \bar{L}_D}_{H_D \bar{H}_B}\right\rangle-\frac{1}{2}\begin{pmatrix}\left\|\Psi^{\bar{L}_D \bar{L}_B}_{\bar{H}_D \bar{H}_B}\right\rangle-\left\|\Psi^{L_D \bar{L}_B}_{H_D \bar{H}_B}\right\rangle\\-\left\|\Psi^{\bar{L}_D L_A}_{\bar{H}_D H_B}\right\rangle+\left\|\Psi^{L_D L_B}_{H_D H_B}\right\rangle\end{pmatrix}\right)$
$\left\|D^{+-}B^{+-}A\right\rangle_{CSP}$	$\frac{1}{2}\left(\left\|\Psi^{\bar{L}_D \bar{L}_B}_{\bar{H}_D \bar{H}_B}\right\rangle+\left\|\Psi^{L_D \bar{L}_B}_{H_D \bar{H}_B}\right\rangle+\left\|\Psi^{\bar{L}_D L_B}_{\bar{H}_D H_B}\right\rangle+\left\|\Psi^{L_D L_B}_{H_D H_B}\right\rangle\right)$
$\left\|DB^{+-}A^{+-}\right\rangle^{SA}_{CTP}$	$\sqrt{\frac{1}{3}}\left(\left\|\Psi^{\bar{L}_B L_A}_{\bar{H}_A H_B}\right\rangle+\left\|\Psi^{L_B \bar{L}_A}_{H_A \bar{H}_B}\right\rangle-\frac{1}{2}\begin{pmatrix}\left\|\Psi^{\bar{L}_A \bar{L}_B}_{\bar{H}_A \bar{H}_B}\right\rangle-\left\|\Psi^{L_A \bar{L}_B}_{H_A \bar{H}_B}\right\rangle\\-\left\|\Psi^{\bar{L}_A L_A}_{\bar{H}_A H_B}\right\rangle+\left\|\Psi^{L_A L_B}_{H_A H_B}\right\rangle\end{pmatrix}\right)$
$\left\|DB^{+-}A^{+-}\right\rangle^{SA}_{CSP}$	$\frac{1}{2}\left(\left\|\Psi^{\bar{L}_A \bar{L}_B}_{\bar{H}_A \bar{H}_B}\right\rangle+\left\|\Psi^{L_A \bar{L}_B}_{H_A \bar{H}_B}\right\rangle+\left\|\Psi^{\bar{L}_A L_B}_{\bar{H}_A H_B}\right\rangle+\left\|\Psi^{L_A L_B}_{H_A H_B}\right\rangle\right)$
$\left\|D^{-}B^{+}A^{+-}\right\rangle^{SA}_{CTP}$	$\sqrt{\frac{1}{3}}\left(\left\|\Psi^{\bar{L}_D L_A}_{\bar{H}_B H_D}\right\rangle+\left\|\Psi^{L_D \bar{L}_A}_{H_B \bar{H}_D}\right\rangle-\frac{1}{2}\begin{pmatrix}\left\|\Psi^{\bar{L}_A \bar{L}_D}_{\bar{H}_B \bar{H}_D}\right\rangle-\left\|\Psi^{L_A \bar{L}_D}_{H_B \bar{H}_D}\right\rangle\\-\left\|\Psi^{\bar{L}_A L_D}_{\bar{H}_B H_D}\right\rangle+\left\|\Psi^{L_A L_D}_{H_B H_D}\right\rangle\end{pmatrix}\right)$
$\left\|D^{-}B^{+}A^{+-}\right\rangle^{SA}_{CSP}$	$\frac{1}{2}\left(\left\|\Psi^{\bar{L}_A \bar{L}_D}_{\bar{H}_B \bar{H}_D}\right\rangle+\left\|\Psi^{L_A \bar{L}_D}_{H_B \bar{H}_D}\right\rangle+\left\|\Psi^{\bar{L}_A L_D}_{\bar{H}_B H_D}\right\rangle+\left\|\Psi^{L_A L_D}_{H_B H_D}\right\rangle\right)$
$\left\|D^{-}B^{+-}A^{+}\right\rangle_{CTP}$	$\sqrt{\frac{1}{3}}\left(\left\|\Psi^{\bar{L}_D L_B}_{\bar{H}_A H_{D(A)}}\right\rangle+\left\|\Psi^{L_D \bar{L}_B}_{H_A \bar{H}_{D(A)}}\right\rangle-\frac{1}{2}\begin{pmatrix}\left\|\Psi^{\bar{L}_B \bar{L}_D}_{\bar{H}_A \bar{H}_D}\right\rangle-\left\|\Psi^{L_B \bar{L}_D}_{H_A \bar{H}_D}\right\rangle\\-\left\|\Psi^{\bar{L}_B L_D}_{\bar{H}_A H_D}\right\rangle+\left\|\Psi^{L_B L_D}_{H_A H_D}\right\rangle\end{pmatrix}\right)$
$\left\|D^{-}B^{+-}A^{+}\right\rangle_{CSP}$	$\frac{1}{2}\left(\left\|\Psi^{\bar{L}_B \bar{L}_D}_{\bar{H}_A \bar{H}_D}\right\rangle+\left\|\Psi^{L_B \bar{L}_D}_{H_A \bar{H}_D}\right\rangle+\left\|\Psi^{\bar{L}_B L_D}_{\bar{H}_A H_D}\right\rangle+\left\|\Psi^{L_B L_D}_{H_A H_D}\right\rangle\right)$

[α] First column: mathematical notation for the spin-adapted many-electron basis states. Second column: spin-spatial multi-electronic states as linear combinations of doubly excited Slater determinants (CTP: correlated Triplet-Pair; CSP: correlated Singlet-Pair).

2. Hamiltonian Matrix Elements

2.1 Diagonal Matrix Elements

In the table below, we give exact expressions for the diagonal elements ${}^{SA}\langle \Psi_n | \hat{H}^{el} | \Psi_n \rangle^{SA}$ of the Hamiltonian $\hat{H}^{el} = \hat{h}^{1e} + \hat{V}^{2e}$. Each element is given as a function of ionization potentials (IP), electron affinities (EA), core terms, Coulomb and exchange integrals, calculated using the Slater-Cordon rules in the above-mentioned basis of states. [4,10]

In our computations we use these exact expressions to compute the Hamiltonian for the reference systems and for the coupling plots. In particular, we compute the 1e and 2e contributions in each equation of Table S3 using the GAMESS-US [11-13] program in the fragment-orbital representation. The main text presents approximate expressions for the lowest-lying states (Table 1 in the main text). The validity of each approximate expression is verified from the ab initio computations of the different contributions in the exact expressions shown in Table S3.

In the equations of Table S3, $\hat{V}_{e-n_k}$ denote Coulombic attractive interactions between the electrons and the kth fragment nuclei (part of the core term in the Hamiltonian). $J_{P_i Q_j}$ and $K_{P_i Q_j}$ are Coulomb and exchange 2e integrals involving the ψ_{P_i} and ψ_{Q_j} fragment molecular orbitals (MOs), where $P,Q = H,L$ and $i,j = D,B,A$, [4]

$$J_{P_i Q_j} \equiv \left(P_i P_i \middle| Q_j Q_j\right) = \int d^3\vec{r}_1\, d^3\vec{r}_2\, \psi^*_{P_i}(\vec{r}_1)\, \psi_{P_i}(\vec{r}_1)\, r^{-1}_{12}\, \psi^*_{Q_j}(\vec{r}_2)\, \psi_{Q_j}(\vec{r}_2), \qquad [S1]$$

$$K_{P_iQ_j} \equiv \left(P_i\, Q_j \middle| Q_j P_i\right) = \int d^3\vec{r}_1\ d^3\vec{r}_2\ \psi^*_{P_i}\left(\vec{r}_1\right) \psi_{Q_j}\left(\vec{r}_1\right) r^{-1}{}_{12}\ \psi^*_{Q_j}\left(\vec{r}_2\right) \psi_{P_i}\left(\vec{r}_2\right). \qquad \text{[S2]}$$

Table S3. Examples of Analytical Expressions for the Basis-State Energies (Diagonal Elements of the Many-Electron Hamiltonian) $^{\alpha}$

**State**	**char.**	$^{SA}\langle \boldsymbol{\Psi_n} \vert \boldsymbol{\hat{H}^{el}} \vert \boldsymbol{\Psi_n} \rangle^{SA}$
	**Singly Excited States**	
$\left\vert D^{+-} B A\right\rangle^{SA}$	LE (in)	$IP^D - EA^D - J_{H_DL_D} + 2K_{H_DL_D}$ $+\langle L_D \vert \hat{V}_{e-n_A} \vert L_D\rangle + \langle L_D \vert \hat{V}_{e-n_B} \vert L_D\rangle - \langle H_D \vert \hat{V}_{e-n_A} \vert H_D\rangle - \langle H_D \vert \hat{V}_{e-n_B} \vert H_D\rangle$ $+2J_{H_AL_D} + 2J_{H_BL_D} - 2J_{H_DH_A} - 2J_{H_DH_B}$ $-K_{H_AL_D} - K_{H_BL_D} + K_{H_DH_A} + K_{H_DH_B}$
$\left\vert D^{+} B^{-} A\right\rangle^{SA}$	CT (B-)	$IP^D - EA^B$ $+\langle L_B \vert \hat{V}_{e-n_D} \vert L_B\rangle + \langle L_B \vert \hat{V}_{e-n_A} \vert L_B\rangle - \langle H_D \vert \hat{V}_{e-n_A} \vert H_D\rangle - \langle H_D \vert \hat{V}_{e-n_B} \vert H_D\rangle$ $+J_{H_DL_B} + 2J_{H_AL_B} - 2J_{H_DH_B} - 2J_{H_DH_A}$ $+K_{H_DL_B} - K_{H_AL_B} + K_{H_DH_B} + K_{H_DH_A}$
$\left\vert D^{-} B^{+} A\right\rangle^{SA}$	CT (B+)	$IP^B - EA^D$ $+\langle L_D \vert \hat{V}_{e-n_B} \vert L_D\rangle + \langle L_D \vert \hat{V}_{e-n_A} \vert L_D\rangle - \langle H_B \vert \hat{V}_{e-n_A} \vert H_B\rangle - \langle H_B \vert \hat{V}_{e-n_D} \vert H_B\rangle$ $+J_{H_BL_D} + 2J_{H_AL_D} - 2J_{H_DH_B} - 2J_{H_BH_A}$ $+K_{H_BL_D} - K_{H_AL_D} + K_{H_DH_B} + K_{H_BH_A}$
$\left\vert D^{-} B A^{+}\right\rangle^{SA}$	CT (DAE)	$IP^A - EA^D$ $+\langle L_D \vert \hat{V}_{e-n_A} \vert L_D\rangle + \langle L_D \vert \hat{V}_{e-n_B} \vert L_D\rangle - \langle H_A \vert \hat{V}_{e-n_B} \vert H_A\rangle - \langle H_A \vert \hat{V}_{e-n_D} \vert H_A\rangle$ $+J_{H_AL_D} + 2J_{H_BL_D} - 2J_{H_DH_A} - 2J_{H_BH_A}$ $+K_{H_AL_D} - K_{H_BL_D} + K_{H_DH_A} + K_{H_BH_A}$
$\left\vert D^{+} B A^{-}\right\rangle^{SA}$	CT (DAE)	$IP^D - EA^A$ $+\langle L_A \vert \hat{V}_{e-n_D} \vert L_A\rangle + \langle L_A \vert \hat{V}_{e-n_B} \vert L_A\rangle - \langle H_D \vert \hat{V}_{e-n_B} \vert H_D\rangle - \langle H_D \vert \hat{V}_{e-n_A} \vert H_D\rangle$ $+J_{H_DL_A} + 2J_{H_BL_A} - 2J_{H_DH_A} - 2J_{H_DH_B}$ $+K_{H_DL_A} - K_{H_BL_A} + K_{H_DH_A} + K_{H_DH_B}$

$\left\vert DB^{+-}A\right\rangle^{SA}$	LE (BE)	$IP^{B}-EA^{B}-J_{H_BL_B}+2K_{H_BL_B}$<br>$+\left\langle L_B\vert\hat{V}_{e-n_A}\vert L_B\right\rangle+\left\langle L_B\vert\hat{V}_{e-n_D}\vert L_B\right\rangle-\left\langle H_B\vert\hat{V}_{e-n_A}\vert H_B\right\rangle-\left\langle H_B\vert\hat{V}_{e-n_D}\vert H_B\right\rangle$<br>$+2J_{H_AL_B}+2J_{H_DL_B}-2J_{H_BH_A}-2J_{H_DH_B}$<br>$-K_{H_AL_B}-K_{H_DL_B}+K_{H_BH_A}+K_{H_DH_B}$
$\left\vert DB^{+}A^{-}\right\rangle^{SA}$	CT (B^+)	$IP^{B}-EA^{A}$<br>$+\left\langle L_A\vert\hat{V}_{e-n_B}\vert L_A\right\rangle+\left\langle L_A\vert\hat{V}_{e-n_D}\vert L_A\right\rangle-\left\langle H_B\vert\hat{V}_{e-n_D}\vert H_B\right\rangle-\left\langle H_B\vert\hat{V}_{e-n_A}\vert H_B\right\rangle$<br>$+J_{H_BL_A}+2J_{H_DL_A}-2J_{H_BH_A}-2J_{H_DH_B}$<br>$+K_{H_BL_A}-K_{H_DL_A}+K_{H_BH_A}+K_{H_DH_B}$
$\left\vert DB^{-}A^{+}\right\rangle^{SA}$	CT (B^-)	$IP^{A}-EA^{B}$<br>$+\left\langle L_B\vert\hat{V}_{e-n_A}\vert L_B\right\rangle+\left\langle L_B\vert\hat{V}_{e-n_D}\vert L_B\right\rangle-\left\langle H_A\vert\hat{V}_{e-n_D}\vert H_A\right\rangle-\left\langle H_A\vert\hat{V}_{e-n_B}\vert H_A\right\rangle$<br>$+J_{H_AL_B}+2J_{H_DL_B}-2J_{H_BH_A}-2J_{H_DH_A}$<br>$+K_{H_AL_B}-K_{H_DL_B}+K_{H_BH_A}+K_{H_DH_A}$
**Doubly Excited States (DE)**		
$\left\vert D^{+-}BA^{+-}\right\rangle^{SA}_{CTP}$	LDE_{CTP} (fi)	$IP^{D}+IP^{A}-EA^{D}-EA^{A}-J_{H_DL_D}-J_{H_AL_A}$<br>$+\left\langle L_D\vert\hat{V}_{e-n_A}\vert L_D\right\rangle+\left\langle L_D\vert\hat{V}_{e-n_B}\vert L_D\right\rangle+\left\langle L_A\vert\hat{V}_{e-n_D}\vert L_A\right\rangle+\left\langle L_A\vert\hat{V}_{e-n_B}\vert L_A\right\rangle$<br>$-\left\langle H_A\vert\hat{V}_{e-n_D}\vert H_A\right\rangle-\left\langle H_A\vert\hat{V}_{e-n_B}\vert H_A\right\rangle-\left\langle H_D\vert\hat{V}_{e-n_A}\vert H_D\right\rangle-\left\langle H_D\vert\hat{V}_{e-n_B}\vert H_D\right\rangle$<br>$-3J_{H_DH_A}-2J_{H_DH_B}-2J_{H_BH_A}+J_{L_DL_A}+J_{H_DL_A}+2J_{H_BL_A}+J_{H_AL_D}+2J_{H_BL_D}$<br>$+\frac{5}{2}K_{H_DH_A}+K_{H_DH_B}+K_{H_BH_A}+\frac{1}{2}K_{L_DL_A}+\frac{1}{2}K_{H_DL_A}-K_{H_BL_A}+\frac{1}{2}K_{H_AL_D}-K_{H_BL_D}$
$\left\vert D^{+}B^{-}A^{+-}\right\rangle^{SA}_{CTP}$	$CTDE_{CTP}$ (B^-)	$IP^{D}+IP^{A}-EA^{B}-EA^{A}-J_{H_AL_A}$<br>$+\left\langle L_B\vert\hat{V}_{e-n_D}\vert L_B\right\rangle+\left\langle L_B\vert\hat{V}_{e-n_A}\vert L_B\right\rangle+\left\langle L_A\vert\hat{V}_{e-n_D}\vert L_A\right\rangle+\left\langle L_A\vert\hat{V}_{e-n_B}\vert L_A\right\rangle$<br>$-\left\langle H_A\vert\hat{V}_{e-n_D}\vert H_A\right\rangle-\left\langle H_A\vert\hat{V}_{e-n_B}\vert H_A\right\rangle-\left\langle H_D\vert\hat{V}_{e-n_A}\vert H_D\right\rangle-\left\langle H_D\vert\hat{V}_{e-n_B}\vert H_D\right\rangle$<br>$-3J_{H_DH_A}-2J_{H_DH_B}-2J_{H_BH_A}+J_{L_BL_A}+J_{H_DL_B}+J_{H_AL_B}+J_{H_DL_A}+2J_{H_BL_A}$<br>$+\frac{5}{2}K_{H_DH_A}+K_{H_DH_B}+K_{H_BH_A}+\frac{1}{2}K_{L_BL_A}-K_{H_DL_B}+\frac{1}{2}K_{H_AL_B}+\frac{1}{2}K_{H_DL_A}-K_{H_BL_A}$
$\left\vert D^{+-}B^{-}A^{+}\right\rangle^{SA}_{CTP}$	$CTDE_{CTP}$ (B^-)	$IP^{D}+IP^{A}-EA^{B}-EA^{D}-J_{H_DL_D}$<br>$+\left\langle L_B\vert\hat{V}_{e-n_D}\vert L_B\right\rangle+\left\langle L_B\vert\hat{V}_{e-n_A}\vert L_B\right\rangle+\left\langle L_D\vert\hat{V}_{e-n_A}\vert L_D\right\rangle+\left\langle L_D\vert\hat{V}_{e-n_B}\vert L_D\right\rangle$<br>$-\left\langle H_D\vert\hat{V}_{e-n_A}\vert H_D\right\rangle-\left\langle H_D\vert\hat{V}_{e-n_B}\vert H_D\right\rangle-\left\langle H_A\vert\hat{V}_{e-n_D}\vert H_A\right\rangle-\left\langle H_A\vert\hat{V}_{e-n_B}\vert H_A\right\rangle$<br>$-3J_{H_DH_A}-2J_{H_BH_A}-2J_{H_DH_B}+J_{L_DL_B}+J_{H_AL_B}+J_{H_DL_B}+J_{H_AL_D}+2J_{H_BL_D}$<br>$+\frac{5}{2}K_{H_DH_A}+K_{H_BH_A}+K_{H_DH_B}+\frac{1}{2}K_{L_DL_B}-K_{H_AL_B}+\frac{1}{2}K_{H_DL_B}+\frac{1}{2}K_{H_AL_D}-K_{H_BL_D}$

$\left\vert D^{+-}B^{+}A^{-}\right\rangle_{CTP}^{SA}$	CTDE$_{CTP}$ (B^{+})	$IP^{D}+IP^{B}-EA^{D}-EA^{A}-J_{H_DL_D}$<br>$+\langle L_D\vert\hat{V}_{e-n_A}\vert L_D\rangle+\langle L_D\vert\hat{V}_{e-n_B}\vert L_D\rangle+\langle L_A\vert\hat{V}_{e-n_D}\vert L_A\rangle+\langle L_A\vert\hat{V}_{e-n_B}\vert L_A\rangle$<br>$-\langle H_D\vert\hat{V}_{e-n_A}\vert H_D\rangle-\langle H_D\vert\hat{V}_{e-n_B}\vert H_D\rangle-\langle H_B\vert\hat{V}_{e-n_D}\vert H_B\rangle-\langle H_B\vert\hat{V}_{e-n_A}\vert H_B\rangle$<br>$-3J_{H_DH_B}-2J_{H_DH_A}-2J_{H_BH_A}+J_{L_DL_A}+J_{H_BL_D}+2J_{H_AL_D}+J_{H_DL_A}+J_{H_BL_A}$<br>$+\frac{5}{2}K_{H_DH_B}+K_{H_DH_A}+K_{H_BH_A}+\frac{1}{2}K_{L_DL_A}+\frac{1}{2}K_{H_BL_D}-K_{H_AL_D}+\frac{1}{2}K_{H_DL_A}-K_{H_BL_A}$
$\left\vert D^{-}B^{+}A^{+-}\right\rangle_{CTP}^{SA}$	CTDE$_{CTP}$ (B^{+})	$IP^{A}+IP^{B}-EA^{A}-EA^{D}-J_{H_AL_A}$<br>$+\langle L_A\vert\hat{V}_{e-n_D}\vert L_A\rangle+\langle L_A\vert\hat{V}_{e-n_B}\vert L_A\rangle+\langle L_D\vert\hat{V}_{e-n_A}\vert L_D\rangle+\langle L_D\vert\hat{V}_{e-n_B}\vert L_D\rangle$<br>$-\langle H_A\vert\hat{V}_{e-n_D}\vert H_A\rangle-\langle H_A\vert\hat{V}_{e-n_B}\vert H_A\rangle-\langle H_B\vert\hat{V}_{e-n_D}\vert H_B\rangle-\langle H_B\vert\hat{V}_{e-n_A}\vert H_B\rangle$<br>$-3J_{H_BH_A}-2J_{H_DH_A}-2J_{H_DH_B}+J_{L_DL_A}+J_{H_BL_A}+2J_{H_DL_A}+J_{H_AL_D}+J_{H_BL_D}$<br>$+\frac{5}{2}K_{H_BH_A}+K_{H_DH_A}+K_{H_DH_B}+\frac{1}{2}K_{L_DL_A}+\frac{1}{2}K_{H_BL_A}-K_{H_DL_A}+\frac{1}{2}K_{H_AL_D}-K_{H_BL_D}$
$\left\vert DB^{+-}A^{+-}\right\rangle_{CTP}^{SA}$	LDE$_{CTP}$ (BE)	$IP^{B}+IP^{A}-EA^{B}-EA^{A}-J_{H_BL_B}-J_{H_AL_A}$<br>$+\langle L_B\vert\hat{V}_{e-n_A}\vert L_B\rangle+\langle L_B\vert\hat{V}_{e-n_D}\vert L_B\rangle+\langle L_A\vert\hat{V}_{e-n_D}\vert L_A\rangle+\langle L_A\vert\hat{V}_{e-n_B}\vert L_A\rangle$<br>$-\langle H_A\vert\hat{V}_{e-n_D}\vert H_A\rangle-\langle H_A\vert\hat{V}_{e-n_B}\vert H_A\rangle-\langle H_B\vert\hat{V}_{e-n_A}\vert H_B\rangle-\langle H_B\vert\hat{V}_{e-n_D}\vert H_B\rangle$<br>$-3J_{H_BH_A}-2J_{H_DH_B}-2J_{H_DH_A}+J_{L_BL_A}+J_{H_BL_A}+2J_{H_DL_A}+J_{H_AL_B}+2J_{H_DL_B}$<br>$+\frac{5}{2}K_{H_BH_A}+K_{H_DH_B}+K_{H_DH_A}+\frac{1}{2}K_{L_BL_A}+\frac{1}{2}K_{H_BL_A}-K_{H_DL_A}+\frac{1}{2}K_{H_AL_B}-K_{H_DL_B}$
$\left\vert D^{-}B^{+-}A^{+}\right\rangle_{CTP}^{SA}$	CTDE$_{CTP}$ (BE)	$IP^{A}+IP^{B}-EA^{D}-EA^{B}-J_{H_BL_B}$<br>$+\langle L_B\vert\hat{V}_{e-n_D}\vert L_B\rangle+\langle L_B\vert\hat{V}_{e-n_A}\vert L_B\rangle+\langle L_D\vert\hat{V}_{e-n_A}\vert L_D\rangle+\langle L_D\vert\hat{V}_{e-n_B}\vert L_D\rangle$<br>$-\langle H_B\vert\hat{V}_{e-n_D}\vert H_B\rangle-\langle H_B\vert\hat{V}_{e-n_A}\vert H_B\rangle-\langle H_A\vert\hat{V}_{e-n_D}\vert H_A\rangle-\langle H_A\vert\hat{V}_{e-n_B}\vert H_A\rangle$<br>$-3J_{H_BH_A}-2J_{H_DH_B}-2J_{H_DH_A}+J_{L_DL_B}+J_{H_AL_B}+2J_{H_DL_B}+J_{H_BL_D}+J_{H_AL_D}$<br>$+\frac{5}{2}K_{H_BH_A}+K_{H_DH_B}+K_{H_DH_A}+\frac{1}{2}K_{L_DL_B}+\frac{1}{2}K_{H_AL_B}-K_{H_DL_B}+\frac{1}{2}K_{H_BL_D}-K_{H_AL_D}$
$\left\vert D^{+}B^{+-}A^{-}\right\rangle_{CTP}^{SA}$	CTDE$_{CTP}$ (BE)	$IP^{D}+IP^{B}-EA^{A}-EA^{B}-J_{H_BL_B}$<br>$+\langle L_B\vert\hat{V}_{e-n_D}\vert L_B\rangle+\langle L_B\vert\hat{V}_{e-n_A}\vert L_B\rangle+\langle L_A\vert\hat{V}_{e-n_D}\vert L_A\rangle+\langle L_A\vert\hat{V}_{e-n_B}\vert L_A\rangle$<br>$-\langle H_B\vert\hat{V}_{e-n_D}\vert H_B\rangle-\langle H_B\vert\hat{V}_{e-n_A}\vert H_B\rangle-\langle H_D\vert\hat{V}_{e-n_A}\vert H_D\rangle-\langle H_D\vert\hat{V}_{e-n_B}\vert H_D\rangle$<br>$-3J_{H_DH_B}-2J_{H_BH_A}-2J_{H_DH_A}+J_{L_BL_A}+J_{H_DL_B}+2J_{H_AL_B}+J_{H_BL_A}+J_{H_DL_A}$<br>$+\frac{5}{2}K_{H_DH_B}+K_{H_BH_A}+K_{H_DH_A}+\frac{1}{2}K_{L_BL_A}+\frac{1}{2}K_{H_DL_B}-K_{H_AL_B}+\frac{1}{2}K_{H_BL_A}-K_{H_DL_A}$

[α] First column: mathematical notation of the spin-adapted (SA) many-electron basis states. Second column: names of the different groups of these states for the D-B-A system. Third column: exact expressions for the excitation energies of these states for the D-B-A system as a function of ionization potentials, electron affinities, core terms and two-electron integrals.

2.2 Off-Diagonal Matrix Elements

In the table below, we present off-diagonal elements, among some of the above-mentioned spin-adapted states. As with the diagonal elements, the expressions are evaluated using ab initio computations on the reference systems. In the equations below F_{P_i,Q_j} denote Fock matrix elements between the P_i and Q_j MOs. Also, for simplicity of presentation we do not include terms proportional to $S_{P_iQ_j}\left(P_i \neq Q_j\right)$. The off-diagonal elements of the overlap matrix between orbitals in different fragments computed to be very close to zero ($S_{P_iQ_j} \approx 0$, $P_i \neq Q_j$).

Table S4. Analytical Expressions for Hamiltonian Matrix Elements between Spin-Adapted Basis States [α]

Coupling Notation	States involved	Coupling expression
V_{2e}	LE (in) – LDE_{CTP} (fi)	$\sqrt{3/2}\left(\left(H_D L_A \middle\| H_D H_A\right) - \left(L_D L_A \middle\| H_A L_D\right)\right)$
V_{2e}	LE (in) – LE (BE)	$2\left(H_D L_D \middle\| H_B L_B\right) - \left(L_D L_B \middle\| H_D H_B\right)$
V_{2e}	LE (BE) – LDE_{CTP} (BE)	$\sqrt{3/2}\left(\left(H_B L_{D(A)} \middle\| H_{D(A)} H_B\right) - \left(L_{D(A)} L_B \middle\| H_{D(A)} L_B\right)\right)$
V_{2e}	LE (in) – LDE_{CTP} (BE)	$\sqrt{3/2}\left(\left(H_D L_B \middle\| H_D H_B\right) - \left(L_D L_B \middle\| H_B L_D\right)\right)$
-----	LE (BE) – LDE_{CTP} (fi)	-----

V_{2e}	LDE_{CTP} (BE) – LDE_{CTP} (fi)	$\sqrt{3/2}\left(L_{D(A)}L_B \middle| H_{D(A)}H_B\right)$
V_{1e}	CT (B^+) – LE (in)	$-F_{H_D H_B} + 2\left(H_D L_D \middle| H_B L_D\right) - \left(H_D H_B \middle| L_D L_D\right)$
V_{1e}	CT (B^+) – LE (BE)	$F_{L_{D(A)}L_B} + 2\left(H_B L_B \middle| H_B L_{D(A)}\right) - \left(L_{D(A)}L_B \middle| H_B H_B\right)$
V_{1e}	CT (B^+) – LDE_{CTP} (BE)	$\frac{1}{\sqrt{2}}\begin{pmatrix} F_{H_{D(A)}L_B} + 2\left(H_{D(A)}H_B \middle| H_B L_B\right) \\ -2\left(H_{D(A)}L_{D(A)} \middle| L_{D(A)}L_B\right) + \left(H_{D(A)}L_B \middle| L_{D(A)}L_{D(A)}\right) \end{pmatrix}$
-----	CT (B^+) – LDE_{CTP} (fi)	-----
V_{1e}	CT (B^-) – LE (in)	$F_{L_D L_B} + 2\left(H_D L_D \middle| H_D L_B\right) - \left(L_D L_B \middle| H_D H_D\right)$
V_{1e}	CT (B^-) – LE (BE)	$-F_{H_{D(A)}H_B} + 2\left(H_{D(A)}L_B \middle| H_B L_B\right) - \left(H_{D(A)}H_B \middle| L_B L_B\right)$
V_{1e}	CT (B^-) – LDE_{CTP} (BE)	$\frac{1}{\sqrt{2}}\begin{pmatrix} F_{H_B L_{D(A)}} + 2\left(H_B H_{D(A)} \middle| H_{D(A)}L_{D(A)}\right) \\ -2\left(H_B L_B \middle| L_{D(A)}L_B\right) + \left(H_B L_{D(A)} \middle| L_B L_B\right) \end{pmatrix}$
-----	CT (B^-) – LDE_{CTP} (fi)	-----

[α] First column: coupling notation, V_{1e} denotes coupling dominated by the 1e matrix element (Fock matrix element), while V_{2e} is a coupling dominated entirely by 2e matrix elements. Second column: notation of the states involved in the corresponding coupling. Third column: exact expressions for the off-diagonal matrix elements as a function of Fock matrix elements and 2e integrals ignoring overlap off-diagonal matrix elements.

3. Effective Coupling Analysis

As mentioned in the main text, we explore the situation where the initial photoexcitation creates a D-localized singlet exciton that can be approximated by $|in\rangle \approx |D^{+-}BA\rangle^{SA}$ (eq. 1 and first row in Table 1). The coherent SF process leads to a final state that is approximated by the D-A separated correlated triplet-pair $|fi\rangle \approx |D^{+-}BA^{+-}\rangle_{CTP}^{SA}$ (second row of Table 1). The assumption of a D-localized $|in\rangle$ implies that all other intermediate states (third-to-final rows of Table 1) are off-resonant to $|in\rangle$ (and also to $|fi\rangle$, since it has lower energy than $|in\rangle$). We denote this regime of SF as the coherent tunneling regime. Singlet fission will take place when the initial and final states come to resonance at an energy E_{res}. Using standard projection methods, [14-16] we approximate the effective coupling for the SF process by

$$V_{SF} = \langle fi|\hat{H}^{el}\hat{G}^{(int)}(E_{res})\hat{H}^{el}|in\rangle = \sum_k \frac{\langle fi|\hat{H}^{el}|\tilde{\Psi}_k\rangle\langle\tilde{\Psi}_k|\hat{H}^{el}|in\rangle}{E_{res}+i\delta-\tilde{E}_k}. \quad [S3]$$

In the equation above $E_{res} = (E_{in}+E_{fi})/2$ and

$$\hat{G}^{(int)}(E_{res}) = \left(E_{res}\hat{I}^{(int)} + i\delta - \hat{H}^{(int)}\right)^{-1} \quad [S4]$$

is the Green's function operator for the exact Hamiltonian of the subspace of all intermediate states. That is, $\hat{H}^{(int)} = \hat{Q}^{(int)}\hat{H}^{el}\hat{Q}^{(int)}$, where $\hat{Q}^{(int)} = \hat{I} - (|in\rangle\langle in| + |fi\rangle\langle fi|)$. $\tilde{E}_k$ and $|\tilde{\Psi}_k\rangle$ are the eigenenergies and eigenstates of this Hamiltonian $\left(\hat{H}^{(int)}|\tilde{\Psi}_k\rangle = \tilde{E}_k|\tilde{\Psi}_k\rangle\right)$. The eigenstate expression for V_{SF} enables decomposing the SF coupling into channel contributions from each eigenstate. Using perturbation theory, V_{SF} can be further decomposed into sums of terms each of which

can be interpreted as a SF pathway that contributes to the total SF coupling. Each pathway starts in $|in\rangle$, visits some of the intermediate states and ends in $|fi\rangle$. The validity of eq. S3 in approximating the effective SF coupling is checked via exact diagonalization performed by setting $E_{in} \to E_{res}$ and $E_{fi} \to E_{res}$. In the off-resonant tunneling regime exact diagonalization for resonant $|in\rangle$ and $|fi\rangle$ gives two eigenstates $|\Psi_{\pm}\rangle \approx \frac{1}{\sqrt{2}}(|in\rangle \pm |fi\rangle)$ with energy splitting between them $|E_{+} - E_{-}| \approx 2|V_{SF}|$. As quasi-resonance in approached the two lowest eigenstates have main (equal) contributions from the initial and final states but there is more mixing with the other intermediate states (as compared to the off-resonance case).

We analyse the SF pathways for each set of V_{SF} plots by turning-off off-diagonal matrix elements in the Hamiltonian connecting a specific state with the rest, and observing how the effective coupling is affected. Specifically, we compute the ratio $\eta_{|\Psi_k\rangle}$ of V_{SF} when we turn-off the contribution of a specific intermediate state in the Hamiltonian matrix to the exact V_{SF} value. The higher the ratio $\eta_{|\Psi_k\rangle}$, the greater the contribution of the particular intermediate state to the exact effective coupling. In addition to the above analysis we also derive pathways by doing a perturbative expansion of eqs S3 and S4 in powers of the off-diagonal matrix elements of the Hamiltonian and keeping the highest terms (strongest pathways).

4. Difference among Contour Plots

To explore the relative importance between intermediate-state energies as compared to interstate couplings in determining the $|V_{SF}|$ values we focus on the cases of the π-stacking and non-π-stacking conformations as shown in the Figs 1a, 1b. We produce two different plots $\left|ln|V_{SF}|/|V'_{SF}|\right|$ and $\left|ln|V_{SF}|/|V''_{SF}|\right|$ as a function Y/X and z/X , where V_{SF} is the effective coupling for the non-π-stacking conformation (as in the original Fig. 2b). V'_{SF} is the corresponding value if we replace only the off-diagonal elements of the non-π-stacking system with the ones of the π-stacking system and V''_{SF} is the value when we replace only the diagonal elements.

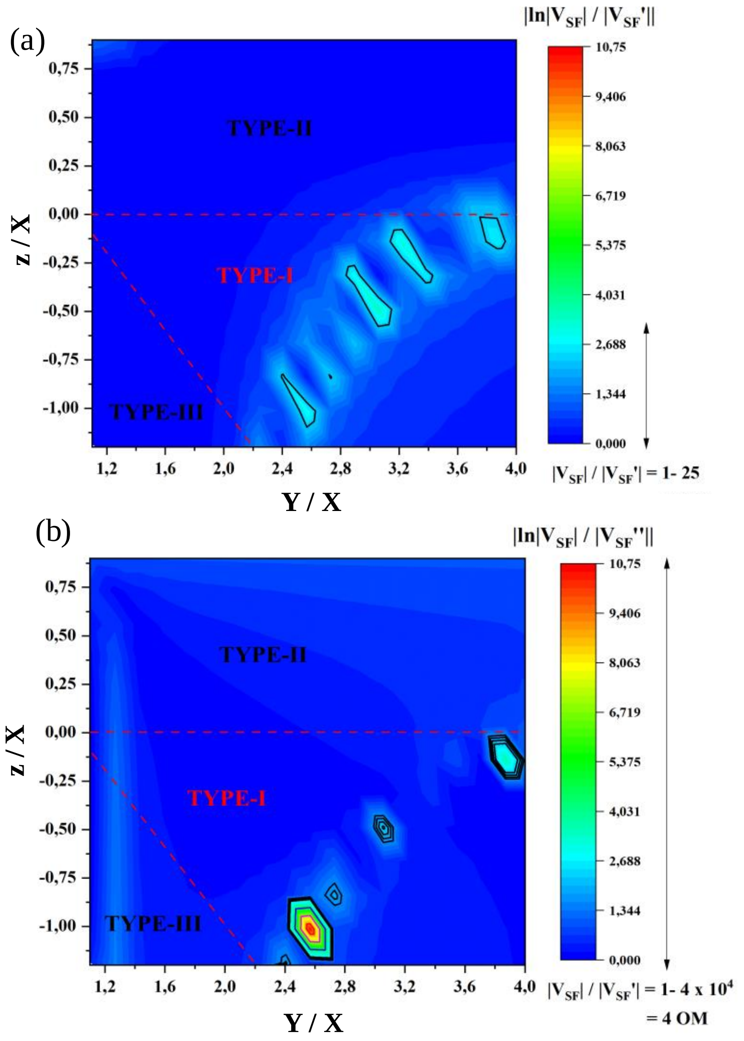

Figure S1. Difference contour plots. (a) $\left|ln|V_{SF}|/|V'_{SF}|\right|$ and (b) $\left|ln|V_{SF}|/|V''_{SF}|\right|$ as a function of Y/X and z/X . The dashed lines outline the three regimes defined in Scheme 2. The black contour corresponds to a coupling ratio equal to 10 which roughly corresponds to a difference of an order of magnitude (OM), the blue to 2 OM, magenta to 3 OM and red to 4 OM. The colormap scaling is the same for both plots.

As can be seen from Fig. S1 the largest change in effective coupling comes from the diagonal Hamiltonian elements, as they can cause a change in effective coupling up to four orders of magnitude (OM). In the case of off-diagonal elements (inter-state couplings) the maximum change is limited to one OM. We find that the magnitudes of the V_{1e} and V_{2e} do not vary significantly among the reference structures of Fig. 1 (maximum percentage changes and average percentage for V_{1e} of the order of 40% and 16%, respectively, and for V_{2e} 33% and 15%, respectively). The maximum coupling magnitude is of the order of $0.1eV$.

5. Generality of the Analytical Model

In the main text and in SI sections 1 and 2, the analytical formulas for energies and couplings are presented in a fragment HOMO-LUMO basis because we want to correlate our analysis with *IPs* and *EAs* that are known for many molecules that may be used as potential fragments (and that are used as indicators of bridge-resonance in the experiments mentioned in text). However, the formulas presented in the Tables S1-S4, from a mathematical point of view, are general (i.e., not restricted to HOMO and LUMO fragment orbitals). We illustrate this with some examples for singly and doubly-excited states.

From Tables S1, S2 consider the spin-adapted singly excited states, $\left|D^{+-}BA\right\rangle^{SA}$ (intrafragment excitation) and $\left|D^{+}B^{-}A\right\rangle^{SA}$ (interfragment excitation) and the spin-adapted doubly excited state that is one of the bridge-mixed CTP states, which we find to be a "bottleneck" state, $\left|D^{+-}B^{+-}A\right\rangle_{CTP}^{SA}$. These formulas are valid even if the fragment hole and electron orbitals are not HOMO and LUMO. Namely, one just needs to replace in the equations H_F, L_F with O_F, V_F where O_F, V_F is an occupied and a virtual orbital, respectively, of fragment F ($F = D, B, A$),

$$\left|D^{+-}BA\right\rangle^{SA} = \frac{1}{\sqrt{2}}\left(\left|\Psi_{O_D}^{V_D}\right\rangle + \left|\Psi_{\bar{O}_D}^{\bar{V}_D}\right\rangle\right), \left|D^{+}B^{-}A\right\rangle^{SA} = \frac{1}{\sqrt{2}}\left(\left|\Psi_{O_D}^{V_B}\right\rangle + \left|\Psi_{\bar{O}_D}^{\bar{V}_B}\right\rangle\right) \ldots \quad \text{[S5]}$$

What these orbitals are is system-specific and should be deduced from experiment coupled with ab initio computations on the system under study.

The corresponding energies and coupling expressions between diabatic states are also general. Again, for the fragment HOMO-LUMO basis some examples are given in Tables S3 and S4. In Table S3, we have written the energies in terms of fragment IPs and EAs. However, if we substitute the equations for each fragment IP and EA in these expressions, e.g., $IP^D = -\left\langle H_D \middle| \hat{h}^{1e} \middle| H_D \right\rangle - J_{H_D H_D}$, $EA^D = -\left\langle L_D \middle| \hat{h}^{1e} \middle| L_D \right\rangle - 2J_{H_D L_D} + K_{H_D L_D}$ etc., we get the energies of Table S3 as functions only of 1e and 2e matrix elements that are derivable from ab initio computations. Having written these energies in terms of 1e and 2e matrix elements we only need to replace H_F, L_F with O_F, V_F to get the generalized expressions. For example, for the first state in eq. S5,

$$E_{|D^{+-}BA\rangle^{SA}} = \langle V_D|\hat{h}^{1e}|V_D\rangle - \langle O_D|\hat{h}^{1e}|O_D\rangle - J_{O_DO_D} + J_{O_DV_D} + K_{O_DV_D}$$
$$+\left\langle V_D\left|\hat{V}_{e-n_A}\right|V_D\right\rangle + \left\langle V_D\left|\hat{V}_{e-n_B}\right|V_D\right\rangle - \left\langle O_D\left|\hat{V}_{e-n_A}\right|O_D\right\rangle - \left\langle O_D\left|\hat{V}_{e-n_B}\right|O_D\right\rangle \quad \text{[S6]}$$
$$+2J_{V_DO_A} + 2J_{V_DO_B} - 2J_{O_DO_A} - 2J_{O_DO_B}$$
$$-K_{V_DO_A} - K_{V_DO_B} + K_{O_DO_A} + K_{O_DO_B}$$

For a CTP state involving fragment orbitals O_D, V_D and O_B, V_B,

$$E_{|D^{+-}B^{+-}A\rangle^{SA}_{CTP}} = \langle V_D|\hat{h}^{1e}|V_D\rangle + \langle V_B|\hat{h}^{1e}|V_B\rangle - \langle O_D|\hat{h}^{1e}|O_D\rangle - \langle O_B|\hat{h}^{1e}|O_B\rangle$$
$$-J_{O_BO_B} - J_{O_DO_D} + J_{O_BV_B} + J_{O_DV_D} - K_{O_DO_D} - K_{O_BV_B}$$
$$+\left\langle V_B\left|\hat{V}_{e-n_A}\right|V_B\right\rangle + \left\langle V_B\left|\hat{V}_{e-n_D}\right|V_B\right\rangle + \left\langle V_D\left|\hat{V}_{e-n_A}\right|V_D\right\rangle + \left\langle V_D\left|\hat{V}_{e-n_B}\right|V_D\right\rangle$$
$$-\left\langle O_D\left|\hat{V}_{e-n_A}\right|O_D\right\rangle - \left\langle O_D\left|\hat{V}_{e-n_B}\right|O_D\right\rangle - \left\langle O_B\left|\hat{V}_{e-n_A}\right|O_B\right\rangle - \left\langle O_B\left|\hat{V}_{e-n_D}\right|O_B\right\rangle \quad \text{[S7]}$$
$$-3J_{O_BO_D} - 2J_{O_AO_B} - 2J_{O_DO_A} + J_{V_BV_D} + J_{O_BV_D} + 2J_{O_AV_D} + J_{V_BO_D} + 2J_{V_BO_A}$$
$$+\frac{5}{2}K_{O_BO_D} + K_{O_AO_B} + K_{O_DO_A} + \frac{1}{2}K_{V_BV_D} + \frac{1}{2}K_{O_BV_D} - K_{O_AV_D} + \frac{1}{2}K_{V_BO_D} - K_{V_BO_A}$$

The same holds for the analytical expressions of the off-diagonal matrix elements between the many-electron states (Table S4), e.g.,

$$_{CTP}^{SA}\left\langle D B^{+-} A^{+-}\left|\hat{H}^{el}\right|D^{+-} B A^{+-}\right\rangle_{CTP}^{SA} = \sqrt{3/2}\left(L_D L_B|H_D H_B\right) \rightarrow \sqrt{3/2}\left(V_D V_B|O_D O_B\right) \quad \text{[S8]}$$

These general formulas allow us to consider cases where the fragment excited states are not intrafragment $O_F \rightarrow V_F$ or interfragment $O_F \rightarrow V_{F'}$ excitations, but rather linear combinations of excitations involving more than a pair of occupied and virtual orbitals. For example, rather than having the $\left|D^{+-} B A\right\rangle^{SA}$ expression of eq. S5, we could have chosen the excitation to be more complex, such as the linear combination of $\left|D^{+-} B A\right\rangle^{SA} = C\frac{1}{\sqrt{2}}\left(\left|\Psi_{O_D}^{V_D}\right\rangle + \left|\Psi_{\bar{O}_D}^{\bar{V}_D}\right\rangle\right) + C'\frac{1}{\sqrt{2}}\left(\left|\Psi_{O'_D}^{V'_D}\right\rangle + \left|\Psi_{\bar{O}'_D}^{\bar{V}'_D}\right\rangle\right)$ with $O'_D = H_D - 1$ and $V'_D = L_D + 1$.

The choice of fragment excited states will depend on the systems considered and on the experimental and computational information we can get for the nature of their excitations (i.e., if they are single excitations or linear combinations of single excitations). Since we have analytical expressions of the Hamiltonian matrix elements in terms of any O_F, V_F orbitals, we can deduce analytically and computationally the relevant minimum model and approximate pathways that describe the specific system. Finally, the exact formulas for the basis states, their energies and their Hamiltonian interactions are not based on any assumption about the strengths of the interactions. Since the effective coupling is computed exactly by diagonalization of the full Hamiltonian at the initial-to-final state resonance (tunneling) energy (see section 3), the method can treat both asymmetric D-B-A systems and strongly-interacting fragments.

References

(1) Pauncz, R. *The Construction of Spin Eigenfunctions: An Exercise Book*; 1st Ed.; Springer, Boston, MA, 2000.

(2) Pauncz, R. *The Symmetric Group in Quantum Chemistry*; CRC Press, Florida, 1995.

(3) Grabenstetter, J. E.; Tseng, T. J.; Grein, F. Generation of genealogical spin eigenfunctions. *Int. J. Quantum Chem.* **1976**, *10 (1)*, 143-149.

(4) Szabo, A.; Ostlund, N. S. *Modern Quantum Chemistry: Introduction to Advanced Electronic Structure Theory*; Dover, Mineola, New York, 1996.

(5) Scholes, G. D. Correlated Pair States Formed by Singlet Fission and Exciton-Exciton Annihilation. *J. Phys. Chem. A* **2015**, *119*, 12699−12705.

(6) Atkins, P.; Friedman, R. *Molecular Quantum Mechanics*; 5th ed.; Oxford Univ. Press, New York, 2011.

(7) Miyata, K.; Conrad-Burton, F. S.; Geyer, F. L.; Zhu, X.-Y. Triplet Pair States in Singlet Fission. *Chem. Rev.* **2019**, *19,* 4261-4292.

(8) Casanova, D. Theoretical Modeling of Singlet Fission. *Chem. Rev.* **2018**, *118,* 7164-7207.

(9) Breen, I.; Tempelaar, R.; Bizimana, L. A.; Kloss, B.; Reichman, D. R.; Turner, D. B. Triplet Separation Drives Singlet Fission after Femtosecond Correlated Triplet Pair Production in Rubrene. *J. Am. Chem. Soc.* **2017**, *139,* 11745-11751.

(10) Slater, J. C. A Simplification of the Hartree-Fock Method. *Phys. Rev.* **1951**, *81,* 385-390.

(11) Dupuis, M.; Spangler, D.; Wendolowski, J. J. *National Resource for Computations in Chemistry Software Catalog*; University of California, Berkeley, CA, Program QG01, 1980.

(12) Schmidt, M. W.; Baldridge, K. K.; Boatz, J. A.; Elbert, S. T.; Gordon, M. S.; Jensen, J. H.; Koseki, S.; Matsunaga, N.; Nguyen, K. A.; Su, S.; *et al.* General Atomic and Molecular Electronic Structure System. *J. Comput. Chem.* **1983**, *14 (11),* 1347-1363.

(13) Advances in electronic structure theory: GAMESS a decade later; Gordon, M. S., Schmidt, M. W., Dykstra, C. E., Frenking, G., Kim, K. S., Scuseria, G. E., Eds.; Theory and Applications of Computational Chemistry: the first forty years; Elsevier, Amsterdam, 2005; pp 1167-1189.

(14) Löwdin, P.-O. Studies in Perturbation Theory. IV. Solution of Eigenvalue Problem by Projection Operator Formalism. *J. Math. Phys.* **1962**, *3 (5)*, 969-982.

(15) Skourtis, S. S.; Beratan, D. N. Theories of Structure-Function Relationships for Bridge-Mediated Electron Transfer Reactions. *Adv. Chem. Phys.* **1999**, *106*, 377-452.

(16) Skourtis, S. S.; Beratan, D. N.; Onuchic, J. N. The two-state reduction for electron and hole transfer in bridge-mediated electron-transfer reactions. *Chem. Phys.* **1993**, *176*, 501-520.
