# Supplementary material for: The Role of Bridge-State Intermediates in Singlet Fission for Donor–Bridge–Acceptor Systems: A Semianalytical Approach to Bridge-Tuning of the Donor–Acceptor Fission Coupling
Source: J Phys Chem Lett. 2022 Jan 20;13(3):939–46. doi: 10.1021/acs.jpclett.1c03700 (PMC9836358; doi:10.1021/acs.jpclett.1c03700)
Supplement: Supplementary file 2 — jz1c03700_si_002.pdf [file jz1c03700_si_002.pdf]

jz-2021-03700e.R1

Name: Peer Review Information for "The Role of Bridge-state Intermediates in Singlet Fission for Donor-bridge-acceptor Systems: A Semi-analytical Approach to Bridge-tuning of the Donor-acceptor Fission Coupling"

First Round of Reviewer Comments

Reviewer: 1

Comments to the Author

The paper by Skourtis proposes an approach to guide search of D-B-A system with tuned singlet fission rate. I currently see two main limitations, which make the scope of the approaches fairly narrow thus preventing publication in JPCLett.

-for the studied cases (four trimers all based on pentacene) it happens that the local T1 in the D, B and A fragments can be described as HOMO-to-LUMO, but this cannot be generalised (by no means in my opinion) to all kind of chromophores, which limits (significantly) the applicability of the semi-empirical protocol proposed. At least

-in the semi-empirical protocol proposed (from end page 21 to end), the energy of the CSP (coupled singlet pairs) D*B*A intermediate, is estimated as the sum of T1 of D and T1 of B. This may be good estimate for intermolecular DB pairs with relatively small interaction but for covalently bonded pairs, specially if they are conjugated, would not be appropriate.

typo:
Page 6, line 1, should be six electrons in six fragment orbitals, not four electrons in six fragments orbitals.

Finally, the citations seem incomplete as the use of frontier molecular orbitals has already been discussed in field of intramolecular singlet fission.

Reviewer: 2

Comments to the Author

Review “The role of bridge-state intermediates in singlet fission for donor-bridge-acceptor systems: A semi-analytical approach to bridge-tuning of the donor-acceptor fission coupling”

The manuscript describes a detailed theoretical analysis of singlet fission in bichromophoric systems that are coupled through a molecular bridge. These systems are vital for the systematic

unraveling of the fundamentals of singlet fission. The author explicit consider the involvement of bridge states which has received only very little attention so far and therefore the manuscript offers interesting new insights that will certainly aid in the explanation of experimental results and in the design of new bichromophoric systems to study SF. There are two small but important issues that I feel should be addressed before the manuscript can be accepted:

- The authors consistently talk about donor-bridge-acceptor systems, twice in the title actually. This gives the impression that they are dealing with asymmetric bichromophoric systems. While this could in principle be the case, the large majority of SF model systems, including the di-pentacene that is analyzed in the manuscript, is symmetric. I feel that the terminology donor-acceptor is a bit misleading in this sense. So, either it should be renamed, or explained in more detail, especially since there is a class of SF systems that rely of donor-acceptor polymers.
- Scheme 1 is not very clear: there are leftovers of letters and incorrectly aligned 'e' in the top left panel. Also, the figure would be clearer and more intuitive if arrows were used to indicate occupations. This would be more in line with common practice and give some indication of the overall spin of the different states. The same is true for the TOC graphic which is almost impossible to understand without reading the paper first.

I would like to add that the manuscript is very clearly written, making a relatively complex theoretical analysis quite easy to follow.

Author's Response to Peer Review Comments:

Dear Editor,

Below please find our responses to the referee comments regarding the submitted manuscript entitled "*The role of bridge-state intermediates in singlet fission for donor-bridge-acceptor systems: A semi-analytical approach to bridge-tuning of the donor-acceptor fission coupling*".

We have addressed all of the referee comments (shown in italics below, each comment followed by our response). We also made changes to the main text and to the Supporting Information (SI) in response to the comments (in the revised main text these changes are highlighted). We thank the referees for their input that helped us improve the presentation.

Referee 1

The paper by Skourtis proposes an approach to guide search of D-B-A system with tuned singlet fission rate. I currently see two main limitations, which make the scope of the approaches fairly narrow thus preventing publication in JPCLett.

Indeed, the paper proposes an approach to guide the search of tuned singlet fission (SF) rates, but it does much more than that. It also describes a many-electron analytical model for D-B-A SF systems. The utility of an analytical model is evident from the impact that the analogous D-A model of Michl had on interpreting and predicting SF interactions. Our contribution in this respect is that we extend this type of model to include a bridge (i.e., its full excitonic structure up to double intra-bridge and donor-bridge, acceptor-bridge excitations). The results are new and the considerable increase in analytical complexity resulting from including the bridge is evident from reading the SI section (e.g., Tables S1-S4). Due to this complexity, we are forced to narrow the focus on specific classes of systems as first examples of applications of the general model. Finally, our analytical results in combination with computation, explain

recent experiments that observe a bridge-resonance effect for SF rates. These experiments see a correlation with D/A and B HOMO-LUMO gaps. We interpret the correlation in terms of particular many-electron intermediate states and of their energies (the latter are not just differences between HOMO and LUMO energies). Therefore, we believe that our results are very broad and significant, thus publishable in JPCLett.

The limitations described by the referee are not limitations of our approach. We explain why below in detail. In response to the referee's comments we also added the relevant information in the main text (pages 4, 6-7, 10 bottom, 17, 18) and in the SI, where we included a new SI section (section 5) titled "Generality of the analytical model".

-for the studied cases (four trimers all based on pentacene) it happens that the local T1 in the D, B and A fragments can be described as HOMO-to-LUMO, but this cannot be generalised (by no means in my opinion) to all kind of chromophores, which limits (significantly) the applicability of the semi-empirical protocol proposed. At least

We completely disagree, the approach is general. We did not discuss its generality in the original text in any detail due to the size restrictions of a letter and because we necessarily focus on initial applications of the approach. Indeed, the analytical formulas for the many-electron basis states, their energies and couplings are presented in a fragment HOMO-LUMO basis of orbitals. However, the formulas presented in the Tables S1-S4 of the SI, from a mathematical point of view, are fully general. This is now discussed in the new SI section 5 where we give examples of the general formulas.

We illustrate below how one can deduce the general formulas from the HOMO/LUMO ones using some examples of singly and doubly-excited states. From Tables S1 and S2 consider the spin-adapted singly-excited states:

$$\left|D^{+-}BA\right\rangle^{SA}=\frac{1}{\sqrt{2}}\left(\left|\Psi^{L_D}_{H_D}\right\rangle+\left|\Psi^{\bar{L}_D}_{\bar{H}_D}\right\rangle\right)\text{ (intra-fragment excitation),}$$

$$\left|D^{+}B^{-}A\right\rangle^{SA}=\frac{1}{\sqrt{2}}\left(\left|\Psi^{L_B}_{H_D}\right\rangle+\left|\Psi^{\bar{L}_B}_{\bar{H}_D}\right\rangle\right)\text{ (inter-fragment excitation),}$$

and the spin-adapted doubly-excited state that is one of the bridge-mixed correlated-triplet-pair states which we find to be a "bottleneck" intermediate:

$$\left|D^{+-}B^{+-}A\right\rangle^{SA}_{CTP}=\sqrt{\frac{1}{3}}\left(\left|\Psi^{\bar{L}_B\,L_D}_{\bar{H}_D\,H_B}\right\rangle+\left|\Psi^{L_B\,\bar{L}_D}_{H_D\,\bar{H}_B}\right\rangle-\frac{1}{2}\left(\begin{matrix}\left|\Psi^{\bar{L}_D\,\bar{L}_B}_{\bar{H}_D\,\bar{H}_B}\right\rangle-\left|\Psi^{L_D\,\bar{L}_B}_{H_D\,\bar{H}_B}\right\rangle\\-\left|\Psi^{\bar{L}_D\,L_A}_{\bar{H}_D\,H_B}\right\rangle+\left|\Psi^{L_D\,L_B}_{H_D\,H_B}\right\rangle\end{matrix}\right)\right).$$

The kets denote $N-$electron Slater determinants of the D-B-A system. The notation $\chi,\bar{\chi}$ denotes spin-spatial orbitals $\chi \rightarrow \chi(\bar{r})\times\alpha$ and $\bar{\chi} \rightarrow \chi(\bar{r})\times\beta$. These formulas are valid even if the relevant fragment hole and electron orbitals are not HOMO and LUMO. Namely, one just needs to replace in the equations H_F, L_F with O_F, V_F where O_F, V_F is an occupied and a virtual orbital, respectively, of fragment F ($F = D, B, A$). For example,

$$\left|D^{+-}BA\right\rangle^{SA} = \frac{1}{\sqrt{2}}\left(\left|\Psi_{O_D}^{V_D}\right\rangle + \left|\Psi_{\bar{O}_D}^{\bar{V}_D}\right\rangle\right),\ \left|D^{+}B^{-}A\right\rangle^{SA} = \frac{1}{\sqrt{2}}\left(\left|\Psi_{O_D}^{V_B}\right\rangle + \left|\Psi_{\bar{O}_D}^{\bar{V}_B}\right\rangle\right) \text{, etc.}$$

Obviously, the identity of the relevant fragment orbitals is system-specific and should be deduced from experiment coupled with ab-initio computations on the system under study. The corresponding state-energies and the coupling expressions between states can also be put into general form (again starting from the HOMO-LUMO basis). For example, consider the energies of two of the above states,

$$\begin{aligned}
E_{\left|D^{+-}BA\right\rangle^{SA}} = {} & IP^{D} - EA^{D} - J_{H_D L_D} + 2K_{H_D L_D} \\
& + \left\langle L_D \middle| \hat{V}_{e-n_A} \middle| L_D \right\rangle + \left\langle L_D \middle| \hat{V}_{e-n_B} \middle| L_D \right\rangle - \left\langle H_D \middle| \hat{V}_{e-n_A} \middle| H_D \right\rangle - \left\langle H_D \middle| \hat{V}_{e-n_B} \middle| H_D \right\rangle \\
& + 2J_{L_D H_A} + 2J_{L_D H_B} - 2J_{H_D H_A} - 2J_{H_D H_B} \\
& - K_{L_D H_A} - K_{L_D H_B} + K_{H_D H_A} + K_{H_D H_B}
\end{aligned}$$

and

$$\begin{aligned}
E_{\left|D^{+-}B^{+-}A\right\rangle^{SA}_{CTP}} = {} & IP^{B} + IP^{D} - EA^{B} - EA^{D} - J_{H_B L_B} - J_{H_D L_D} \\
& + \left\langle L_B \middle| \hat{V}_{e-n_A} \middle| L_B \right\rangle + \left\langle L_B \middle| \hat{V}_{e-n_D} \middle| L_B \right\rangle + \left\langle L_D \middle| \hat{V}_{e-n_A} \middle| L_D \right\rangle + \left\langle L_D \middle| \hat{V}_{e-n_B} \middle| L_D \right\rangle \\
& - \left\langle H_D \middle| \hat{V}_{e-n_A} \middle| H_D \right\rangle - \left\langle H_D \middle| \hat{V}_{e-n_B} \middle| H_D \right\rangle - \left\langle H_B \middle| \hat{V}_{e-n_A} \middle| H_B \right\rangle - \left\langle H_B \middle| \hat{V}_{e-n_D} \middle| H_B \right\rangle \\
& - 3J_{H_B H_D} - 2J_{H_A H_B} - 2J_{H_D H_A} + J_{L_B L_D} + J_{H_B L_D} + 2J_{H_A L_D} + J_{L_B H_D} + 2J_{L_B H_A} \\
& + \frac{5}{2}K_{H_B H_D} + K_{H_A H_B} + K_{H_D H_A} + \frac{1}{2}K_{L_B L_D} + \frac{1}{2}K_{H_B L_D} - K_{H_A L_D} + \frac{1}{2}K_{L_B H_D} - K_{L_B H_A}
\end{aligned}$$

If we substitute the exact equations for the fragment IPs and EAs, e.g., $IP^{D} = -\left\langle H_D \middle| \hat{h}^{1e} \middle| H_D \right\rangle - J_{H_D H_D}$, $EA^{D} = -\left\langle L_D \middle| \hat{h}^{1e} \middle| L_D \right\rangle - 2J_{H_D L_D} + K_{H_D L_D}$ etc., we get equivalent expressions that are functions only of 1e and 2e matrix elements whose values are determined from ab-initio computations. To get the generalized forms, replace in these expressions H_F, L_F with O_F, V_F (see the new section in the SI titled "Generality of the analytical model" for the generalized forms, e.g., eqs S6, S7). The same holds for the analytical formulas of the off-diagonal matrix elements between the many-electron basis states (Table S4), e.g.,

$$_{CTP}^{SA}\left\langle D\,B^{+-}A^{+-}\left|\hat{H}^{el}\right|D^{+-}B\,A^{+-}\right\rangle_{CTP}^{SA}=\sqrt{3/2}\left(L_D L_B|H_D H_B\right)\rightarrow\sqrt{3/2}\left(V_D V_B|O_D O_B\right)$$

Importantly, we can also treat cases where the relevant excited states in fragment basis are not single intra-fragment $O_F \rightarrow V_F$ or inter-fragment $O_F \rightarrow V_{F'}$ excitations, but rather linear combinations of such excitations (involving more than a pair of occupied and virtual orbitals). For example, rather than having $\left|D^{+-}B\,A\right\rangle^{SA}=\frac{1}{\sqrt{2}}\left(\left|\Psi_{O_D}^{V_D}\right\rangle+\left|\Psi_{\bar{O}_D}^{\bar{V}_D}\right\rangle\right)$ with $O_D = H_D$ and $V_D = L_D$, the relevant state could be more complex, such as the linear combination $\left|D^{+-}B\,A\right\rangle^{SA}=C\frac{1}{\sqrt{2}}\left(\left|\Psi_{O_D}^{V_D}\right\rangle+\left|\Psi_{\bar{O}_D}^{\bar{V}_D}\right\rangle\right)+C'\frac{1}{\sqrt{2}}\left(\left|\Psi_{O'_D}^{V'_D}\right\rangle+\left|\Psi_{\bar{O}'_D}^{\bar{V}'_D}\right\rangle\right)$ with $O'_D = H_D - 1$ and $V'_D = L_D + 1$. Since we have analytical expressions of all types of Hamiltonian matrix elements in our basis in terms of any O_F, V_F orbitals, we can include more than a pair of occupied/virtual orbitals per fragment to build the Hamiltonian for the system. We point out that all numerical computations involve the exact diagonalization of the full DBA Hamiltonian involving up to 40 states. This means that our results are valid regardless of the strength of the interactions between fragments (indeed in the quasi-resonant regime the interactions are strong for the systems studied). The pathway diagrams that describe particular intermediates of the SF process are interpretations of the exact SF coupling that identify the most important states (which, if not included in the full Hamiltonian, would give order of magnitude decrease in the SF coupling). Hence our approach is general but we could not have gotten into such detail in the main text.

*-in the semi-empirical protocol proposed (from end page 21 to end), the energy of the CSP (coupled singlet pairs) D*B*A intermediate, is estimated as the sum of T1 of D and T1 of B. This may be good estimate for intermolecular DB pairs with relatively small interaction but for covalently bonded pairs, specially if they are conjugated, would not be appropriate.*

The referee refers to CTP (coupled triplet-pair) states. CSP states lie much higher in energy (table S2) and do not contribute to a strong SF pathway. Equation 5 contains approximate expressions for the energies of "bottleneck" CTPs. These expressions are shown numerically, using ab-initio computations, to be fair estimates of the analytical exact ones (in Table S3) for the systems under study in the off-resonant regime. As pointed out by the referee, it could certainly be the case that for a different set of systems, the exact formulas for the particular states cannot be approximated by eq. 5, due to stronger interactions. However, the exact formulas for the states, their energies and their Hamiltonian interactions (shown in the SI) are not based on any assumptions about the strengths of the interactions. Further, as mentioned above, the exact SF coupling is computed by diagonalization of the full DBA system. Thus, the method can treat strongly-coupled cases too (e.g., the quasi-resonant regime in this paper). For the systems under study we show only the approximate formulas in the main text (eq. 5)

because they are intuitively appealing. No physical insight can be gained by looking at the exact formulas. For example, compare eq. 5 of the main text for the energy of $\left|D^{+-}B^{+-}A\right\rangle_{CTP}^{SA}$ with the exact expression for this energy shown above, $E_{\left|D^{+-}B^{+-}A\right\rangle_{CTP}^{SA}}$. To avoid any confusion, we now state that the approximate formulas in eq. 5 relate to the particular class of systems under study in the off-resonant regime (Figure 3 caption, pages 16, 17) and that the more general ones are in the SI.

The issue of using fragment methods for strongly-coupled and conjugated systems is related to producing fragment orbitals for such systems. This issue is not specific to singlet fission (it is also encountered in electron-transfer energy transfer etc.). There are several approaches to producing localized (fragment) orbitals in covalently-bonded systems. Our method could use any of these types of fragment orbitals.

typo:
Page 6, line 1, should be six electrons in six fragment orbitals, not four electrons in six fragments orbitals.

We have corrected the statement. We mean six electrons (out of N) in six active fragment orbitals (with single and double excitations).

Finally, the citations seem incomplete as the use of frontier molecular orbitals has already been discussed in field of intramolecular singlet fission.

References (1), (2), (3), (19), (23), (28) and (30) in the original manuscript all use frontier orbitals (and the first three are reviews that cite frontier-orbital work). Given the extensive literature on frontier orbitals we are sure to have missed some work but we already have 49 references in total. In order to bring attention to past frontier-orbital work, we now state in the text that the above-mentioned citations also use frontier orbitals (page 4). However, if the referee points to important relevant references we have missed we will include them.

- The authors consistently talk about donor-bridge-acceptor systems, twice in the title actually. This gives the impression that they are dealing with asymmetric bichromophoric systems. While this could in principle be the case, the large majority of SF model systems, including the di-pentacene that is analyzed in the manuscript, is symmetric. I feel that the terminology donor-acceptor is a bit misleading in this sense. So, either it should be renamed, or explained in more detail, especially since there is a class of SF systems that rely of donor-acceptor polymers.

We agree with the referee that this issue may be confusing. Since the proposed method is applicable to asymmetric systems, we opt for giving a more detailed explanation of its applicability to asymmetric systems rather than changing the title.

As we state in the submitted manuscript, for the regimes considered in the paper, SF takes place by through-bridge off-resonant or quasi-resonant tunneling when the initial and final states come to resonance at a tunneling energy E_{res} (the SF rate being $k_{SF} = |V_{SF}|^2 \rho_{FC}$). This energy is well approximated by $\left(E_{in} + E_{fi}\right)/2$ since $E_{in} > E_{fi}$ if the SF reaction is to proceed in the SF direction. In our computations we always set $E_{in} = E_{fi} = E_{res}$ to simulate the initial-to-final-resonance transition state. For symmetric systems E_{fi} is only slightly lower than E_{in} . But our computational method handles equally well the asymmetric case that has a much lower E_{fi}.

Thus, there is nothing in our approach that makes it inapplicable to the study of the asymmetric case. Of course, in the assymetric case the numerical values for the 1e and 2e integrals that enter the diagonal and off-diagonal Hamiltonian matrix elements between diabatic states will differ as compared to the symmetric one, since B will be interacting with D differently as compared to its interaction with A. This may have an effect on the relative strengths of the main pathways, so our particular pathway results apply to the class of systems under study. We have included this discussion in several places in the text (Figure 3 caption, pages 10, 14 and 16, section 3 and new section 5 in SI).

- Scheme 1 is not very clear: there are leftovers of letters and incorrectly aligned 'e' in the top left panel. Also, the figure would be clearer and more intuitive if arrows were used to indicate occupations. This would be more in line with common practice and give some indication of the overall spin of the different states. The same is true for the TOC graphic which is almost impossible to understand without reading the paper first.

Both Scheme 1 and the TOC graphic have been improved as suggested by the referee. The explanation of Scheme 1 in the caption was also extended.

We hope that our explanations and changes to the manuscript have addressed the comments of the referees.

Sincerely,

Spiros S. Skourtis
